# A hybrid deep boosting framework with adaptive label stabilization for SEM-based porosity estimation in fly-ash cement mortar

**DOI:** 10.3389/frai.2026.1766671

**Published:** 2026-02-23

**Authors:** Abdullah, Muhammad Ateeb Ather, José Luis Oropeza Rodríguez, Carlos Guzmán Sánchez-Mejorada

**Affiliations:** 1 Center for Computing Research, Instituto Politécnico Nacional, Mexico City, Mexico; 2 Department of Computer Sciences, Bahria University, Lahore, Pakistan

**Keywords:** cementitious materials, feature–interaction attention, gradient boosting ensemble, hybrid deep learning, materials informatics, porosity estimation, self-supervised learning, SEM image analysis

## Abstract

**Introduction:**

Accurate measurements of porosity of cementitious matrices are critical in pre- Q7 dicting mechanical behavior, durability, and transportation processes. Traditional methods based on SEM, such as manual thresholding, a simple binarization method, and end-to-end convolutional neural network (CNN) regressors, are, however, highly affected by image contrast variation, polishing quality, magnification, and operator bias. To address these limitations, the current article develops a hybrid deep-boosting framework for fully automatic porosity estimation directly from raw backscattered-electron SEM images of fly-ash cement mortar. The key novelty of the proposed approach lies in the adaptive stabilization of porosity labels and the hybrid fusion of deep semantic and handcrafted texture features, which together improve robustness to imaging artifacts, boundary ambiguity, and overfitting.

**Methods:**

Annotation ground-truth porosity is optimized using an Adaptive Porosity Label Stabilizer (APLS) that successively improves Otsu threshold masks, first using entropy measures and morphological consistency measures to reduce label noise. Multiscale semantic representations are learned on a ResNet-18 backbone, which is trained with SimCLR on SEM data, while local statistical texture is captured using handcrafted gray-level cooccurrence map (GLCM) features. The resulting mismatched set of features is combined with a learnable Hybrid Feature Refinement Block (HFRB) together with a Feature-Interaction Attention (FIA) block, which explicitly characterizes inter-scale relationships among convolutional channels and texture regressors. The latent representation is then condensed and regressed using a weighted ensemble including CatBoost, XGBoost, and LightGBM learners.

**Results and discussion:**

The proposed methodology achieves R^2^ = 0.9816, RMSE = 0.0236, and MAE = 0.00875 on a rigorously held out test set, outperforming baseline methods that rely exclusively on CNN features, handcrafted descriptors, or naïve hybrid combinations. The validity, stability, and physical plausibility of the model are ensured through a comprehensive assessment, including ablation studies, domain-shift experiments, uncertainty and stability calibration, and a hybrid explainability framework (Grad-CAM++, SHAP). The architecture does not require any manual segmentation, generalizes across magnifications and imaging conditions, and provides transparent, domain-consistent explanatory visualizations. Overall, the proposed framework represents an important step toward fast, reliable, and scalable SEMbased porosity estimation in cementitious systems.

## 1 Introduction

Porosity is the ratio between the volume of a porous material that is empty and the total volume of the solid material and is an important microstructural parameter that has a strong effect on mechanical strength, transport, including permeability and diffusivity, and long-term stability of porous media (Holzer et al., 2023; Regalla and Senthil Kumar, 2025). Cementitious materials such as concrete and mortar have a relatively large porosity, thus allowing the ingress of aggressive species, such as chlorides, sulfates, and carbon dioxide, which promotes the mechanism of degradation, such as reinforcement corrosion, freeze–thaw damage, and alkali-silica reaction (Ma et al., 2025; Subash and Subash, 2024). Porosity cannot be properly predicted by accurate measurement to aid in predicting life service, maximizing mix design, and guaranteeing structural integrity of civil infrastructure (Abousnina et al., 2025).

A similar level of criticality can be observed in other porous networks, such as geological structures (rocks and soils), energy materials like battery electrodes, where the flow of fluids, storage capacity, and electrochemical activity are controlled by porosity. In the past, porosity of cement-based materials has been measured in the laboratory through mercury intrusion porosimetry (MIP), gas adsorption, water absorption, or through direct physical measurements (Wang and Zhou, 2022). Despite its precision, such methodologies are devastating, slow, and only give bulk averages that have no spatial resolution (Xia et al., 2024). The advent of non-destructive imaging alternatives, especially scanning electron microscopy (SEM) in backscattered electron (BSE) mode, has become useful in microstructural imaging on the micrometer scale (Ali et al., 2023).

SEM-BSE images take advantage of the atomic-number contrast needed to distinguish pores (in the form of dark) and denser phases, i.e., unhydrated cement, hydration products, and aggregates (Shamaki, 2021). As required to retrieve quantitative porosity through such grayscale micrographs, image-processing pipelines typically comprise preprocessing (e.g., contrast enhancement), segmentation, and threshold-based binarization (Krig, 2025; Kazak et al., 2021). The Otsu thresholding algorithm, which is the most popular, finds the minimum interclass variance of the image histogram in order to mark the difference between pores and solid mass, and has been used to produce ground-truth labelling of porosity in many studies (Hayatdavoudi et al., 2025).

Although being simple and efficient, Otsu thresholding and general image analysis are susceptible to imaging conditions, including magnification, beam energy, quality of the sample polishing, and fluctuations of the contrast (Jastrzębska and Piwowarczyk, 2023). Microstructural complexity is enhanced in heterogeneous systems with additional cementitious material (SCM) like fly ash, slag, or silica fume, as the pozzolanic reaction produces finer pores and denser gel phases, and thus segmentation becomes unreliable (Li et al., 2022). Often, manual intervention or empirical adjustment is necessary, which adds operator bias and makes it only scalable to large datasets (Rouzrokh et al., 2022).

These restrictions have motivated studies in data-driven methods applying machine learning (ML) to predict porosity and automate it using raw images (Nabipour et al., 2025). Modern developments in the field of computer vision and ML have transformed the microstructural characterization of materials science (Holm et al., 2020). CNNs have been very effective in semantic segmentation of SEM and X-ray computed tomography (CT) images, allowing pixel-by-pixel visualization of pores, hydration products, and aggregates (Tang, 2023). U-Net architectures and variants have demonstrated segmentation accuracies beyond 94% of multi-phase concrete microstructures, allowing the individual determination of porosity, degree of hydration, and phase volumes (Yu et al., 2025).

It has also been applied to predict porosity or permeability directly using end-to-end CNN regressors based on 2D/3D images without explicit segmentation (Temani and Feng, 2025). The idea of transfer learning, whereby the pretrained network (e.g., ResNet or EfficientNet trained on ImageNet) is fine-tuned on data with material-specific details, has been particularly useful due to the relative lack of labeled microscopy images compared to natural photographs (Jiang, 2022).

Similar advances in ensemble learning, especially gradient-boosting models like XGBoost, LightGBM, and CatBoost, have set new standards of tabular and structured regression in the engineering world (Ahn et al., 2023). At once, tree-based solutions are effective for nonlinear relations, and interaction between features, as well as noisy data, and they inherently provide robustness by sequentially correcting errors (Blørstad, 2023). In materials informatics, boosting ensembles are used to forecast properties using compositional input (mix proportions) or extracted image features, commonly substantially more accurate than individual models (Li and Zheng, 2023).

However, the majority of prediction-by-image porosity methods are either based on pipelines that are fully deep-learned (a risk of overfitting with small datasets and potentially missing the same hierarchical semantic patterns that deep networks can find) or introduce hand-crafted features [gray-level cooccurrence matrix (GLCM), Haralick textures, local binary patterns], which do not achieve the same hierarchical semantic patterns as found by deep networks (Rabbani et al., 2021). Balanced solutions. A hybridization, combining inductive biases of CNNs to learn the high-level representations, with domain-specific explanatory biases of handcrafted metrics, provides a solution. Hybrid methods are successful in other microstructure tasks, including phase classification and defect detection, but their role in direct porosity regression in cementitious systems has not been studied extensively (Trigka and Dritsas, 2025; Zhang et al., 2024; Barashok et al., 2025).

Although mercury intrusion porosimetry (MIP) is widely used to estimate total porosity in cementitious materials, it primarily probes pore throats and is biased toward larger, percolating pore networks, yielding bulk porosity statistics rather than spatially resolved morphology. Comparative studies have shown systematic discrepancies between MIP and image-based porosity characterization in the small-pore regime, particularly for gel pores and partially connected capillary pores. Using micro-CT and SEM imaging combined with deep learning, Saseendran et al. (2024) and Saseendran et al. (2023) demonstrated that fine-scale porosity and phase connectivity cannot be reliably captured by bulk intrusion techniques. Consequently, MIP measurements are best interpreted as complementary macroscopic indicators rather than pixel-level ground truth. In this work, we therefore focus on SEM-based, spatially resolved porosity estimation, treating laboratory bulk measurements (including MIP) only as auxiliary validation references.

In order to fill these gaps, this study proposes a hybrid machine learning model that can estimate the porosity of fly-ash-modified cement mortar in a fast, accurate, and automated manner based on raw SEM-BSE images. A collection of high-resolution micrographs was curated, and the dataset exhibits diversity in three respects: (i) imaging magnification (200×−1,000×), (ii) porosity range (from 0.02 to 0.98 across samples), and (iii) preparation variability (different polishing/curing batches). This diversity increases the model’s exposure to realistic acquisition variation and is reflected in the domain-shift experiments. The feature extraction was done dually (i) deep embeddings, which were obtained by averaging the pooling layer of a trained ResNet18, that is, hierarchical visual patterns, which can be transferred across natural images; (ii) classical GLCM-derived Haralick textures, coupled with first-order statistics and local binary patterns, which encode fine-scale microstructural cues to pore-solid contrast. The resulting concatenated feature was then input into a series of state-of-the-art gradient-boosting regressors, namely XGBoost, LightGBM, and CatBoost, whose predictions were all averaged to produce the final output. The hybrid model had an *R*-squared equal to 0.982, RMSE equal to 0.0237, and MAE equal to 0.0087, which is significantly higher than the results of deep feature, handcrafted features, and individual boosting feature-only baseline models.

Recent studies demonstrate that fully deep-learning approaches, such as convolutional autoencoders and deep neural networks, can achieve accurate phase and porosity segmentation in cementitious materials when sufficient labeled data and careful regularization are available (Sheiati et al., 2023; Sheiati et al., 2022). However, SEM datasets in cementitious research are often limited in size and affected by contrast variability, polishing artifacts, and boundary ambiguity, which can increase sensitivity to overfitting and domain shift. To address these practical constraints, the present work adopts a hybrid strategy that integrates deep feature extraction with handcrafted morphological descriptors and ensemble regression to enhance robustness and generalization under limited-data conditions.

The complementary utility of hybrid features and ensemble diversity can be proved with the help of ablation studies. The given method requires little preprocessing, is resistant to a wide range of imaging artefacts, and does not require per-image thresholding or segmentation. This study has three main contributions. To begin with, we propose a novel predictive, end-to-end, segmentation-free, porosity estimator on the scanning electron microscopy (SEM) images, where hybrid features of deep-learning and handcrafted characteristics are incorporated to achieve the state-of-the-art performance of fly-ash cement mortar systems. Second, we report the effectiveness of a voting assembly of XGBoost, LightGBM, and CatBoost, thus highlighting the merits of gradient-boosting algorithms in the process of microstructure-property regression. Third, we offer a complete reproducible, open-source workflow, i.e., its source code and pretrained models, to be extended to other cementitious compositions, added cementitious materials (SCMs), or microstructural descriptors like pore size distribution and connectivity.

## 2 Related work

Three-dimensional microstructural characterization has become the pillar in the description of the behaviors of cement-based composites. Specifically, the X-ray microtomography has become unavoidable given the ability to resolve objects less than one micron. Similar experiments have been used at synchrotron centers like the European Synchrotron Radiation Facility (ESRF) and observed cement hydration, pore connectivity, and phase distributions in detail. A comprehensive database of these data is in the public domain, such as the Visible Cement Data Set, and thus makes it easy to identify, separate, and model associations between transport and property according to volumetric microstructure. However, with these developments, the use of high-resolution three-dimensional imaging to predict mechanical behaviors at a continuum of mix parameters is under-researched, which forms a conspicuous gap in the correlation between microstructure and macroscopic behaviors (Kazemi et al., 2022). Equivalent studies have determined the effect of cement strength classification, mixed proportions, and curing regimes on porosity development and strength progression, and the functions of hydration products and pore-structure have been described using scanning electron microscopy (SEM) and energy-dispersive X-ray spectroscopy (EDS). Artificial neural network (ANN) models have already demonstrated significant potential in predicting mechanical performance in a variety of conditions, but there is less evidence of success in combining experimental multi-scale characterization with multi-objective ANN modelling, highlighting the importance of more microstructure-performance models (Qian et al., 2022).

Research on SEM analysis has hitherto been conducted mostly on segmentation, measurement of hydration products, and phase measurements. Traditional methods of image processing (thresholding, edge detection, and manual feature extraction, etc.) are still common but are often criticized as subjective and sensitive to imaging variations. Recent advances in convolutional neural networks (CNNs) and transfer-learning methods have enormously contributed to the classification of images in relation to concrete diagnostics, but the list of studies that aim to classify concrete mixtures in terms of SEM micrographs is small. This has caused an urgent need to develop automated, scalable deep-learning models specifically trained on cementitious materials (Qian et al., 2023). Further limitations have been found in segmentation tasks: even though methods such like k k-k-nearest neighbors (KNN), support-vector machines (SVM), and early deep-learning architectures have been optimized to encourage automation, most of these algorithms were initially trained on more general datasets and do not target a specific microstructure. This deficiency highlights the need to have full automation and deep learning-based segmentation, which are capable of facilitating quantitative analysis of cement paste (Wang et al., 2023).

The macroscopic evidence, including color, weakening strength, and ultrasonic velocity, cannot be relied upon in fire-damaged concrete, as they are very sensitive to the mix composition. The experiments conducted by SEM have shown that temperature-dependent changes in the hydration products can be observed, yet conventional segmentation and classical machine-learning approaches are unable to identify such intricate characteristics. Despite the promise of CNNs in structural damage imaging, few studies have endeavored to directly estimate fire histories in terms of temperature based on the SEM microstructures; thus, they provide a significant research gap (Li et al., 2023a). On the same note, research on cementitious materials that involve seawater has focused mainly on the macro-performance, with very little research being done on the micro-level of microstructural development. Although the SEM-EDS mapping and clustering methods improve the phase detection, the current problems associated with noise, overlap of grey-scale values, and computational cost are overpowering, thus requiring the implementation of more advanced methods of characterization (Radica et al., 2024).

Two-dimensional imaging modalities have limited comparative studies. Even though optical microscopy, SEM, and X-ray-based techniques are widely used, there is a very limited systematic evaluation of the accuracy, cost, and efficiency of these techniques in construction materials. The phase abundance or grain-size distributions that have been extensively studied in the existing literature are mostly quantified without combining various imaging modalities. This gap testifies to the importance of benchmarking the research to increase textural analysis of mortars and other materials (Sobuz et al., 2024). The biochar and other supplementary materials have been studied in sustainability-based research that has found improvements in the hydration kinetics, fracture performance, and permeability. ANN, adaptive neuro-fuzzy inference systems (ANFIS), and regression methods are machine-learning algorithms applicable to strength prediction; however, no comparative analysis of these procedures has been carried out. There is a lack of research, particularly focusing on the wood-sawdust biochar together with multimodal machine-learning prediction, which gives additional motivation to research in this area.

Complementary studies have investigated calorimetry and SEM-based studies on hydration and have found inefficiency of heat-release models at old age and possibilities of combining microstructural and calorimetric data to characterize long-term hydration (Attari et al., 2016). Studies on sulfate attack have discovered the formation of ettringite and monosulfate (MSA) consumption as the major deterioration process, whereas slag, pozzolans, and limestone produce different effects on the resistance and durability. SEM–EDX imaging and supervised classification are becoming more effective in the optimization of mineralogical mapping, but quantitative analysis of MSA pockets distribution and buffering behavior is insufficient (Meulenyzera et al., 2015). The microstructural characterization of SEM-BSE and micro-CT has also been introduced through the use of deep learning, but the comparative convolutional neural network-based analysis of the imaging modalities is yet to be determined (Bentz et al., 2002).

The nanoindentation studies have further improved micromechanical understanding of cement phases, especially in the interfacial transition zone (ITZ). Phase clustering has been enabled by machine-learning methods using indentation data, but integrated approaches between unsupervised and supervised are currently being developed (Wu et al., 2026). The agro-waste and nanoparticles, such as wood ash, fly ash, and nano-TiO 2, have been investigated concerning the effects of additives, and ANN-based prediction of the strength has been applied at high temperatures, but the interactions of green-synthesized wood ash and nano-TiO 2 have not been studied thoroughly (Koumoulos et al., 2019). Nanosilica dispersion, the inability of SEM-EDS to detect nanoparticles, and the possible method of segmentation, e.g., edXIA, have been studied by other researchers; however, evaluating the dispersion of nanoparticles in hardened material is difficult (Raheem et al., 2023). There is a lack of chemo-mechanical research, which combines SEM-EDS, nanoindentation, and homogenization models, although they have the potential to provide breakthroughs in unsupervised, chemically resolved data segmentation (Sargam and Wang, 2021).

The micro-CT characterization of microcapsule-based self-healing systems with the help of machine learning has potential, but capsules and pores cannot be distinguished due to similar grayscale (Maragh et al., 2021). Later developments in EDS segmentation, including the use of K-means, the use of SVM-MRF, GMM, guided filtering, and SLIC superpixels, have improved phase interpretation. However, these algorithms remain based on expert measures of metrics, which require interpretable clustering and dimensionality reduction (Wang et al., 2022). Data-driven models of material behavior, connecting microstructural parameters derived by SEM with compressive strength, highlight the strength of this data-driven method of modeling complex material behavior that conventional models fail to describe. Research on nano-TiO 2 also confirms the necessity of multiscale characterization: TiO 2 regulates the hydration rate, the optimization of nano and meso scales pore structure, and resistance against wear and tear. However, the processes that facilitate such improvements, especially how these nano-scale changes that are caused by TiO 2 can be translated into macro-scale durability effects, have not been fully comprehended (Shafaei et al., 2020).

Parallel developments in high-resolution microtomography have made it possible to compare SEM observations with Micro-CT, providing researchers with a chance to test or reject long-standing assumptions about microstructural characteristics in hardened cement paste, such as its patchy structure. Micro-CT is complementary to SEM because it provides nondestructive 3D data of pores, aggregates, and hydration products, thereby removing the ambiguities caused by sample-preparation artefacts and 2D projection limits. These parallel imaging studies suggest the need to use multimodal validation to obtain the right microstructural interpretation. Altogether, the development of ANN-based predictions of properties, nano-TiO 2 scales, and cross-modal imaging verification forms the initial groundwork of modern microstructure-property studies. At the same time, they reveal the complexity of issues linked with the creation of fully integrated, automated, and mechanistically based characterization systems (Li et al., 2023b; Onal and Ozturk, 2010; Diamond and Landis, 2007).

## 3 Materials and methods

**Figure 1:**
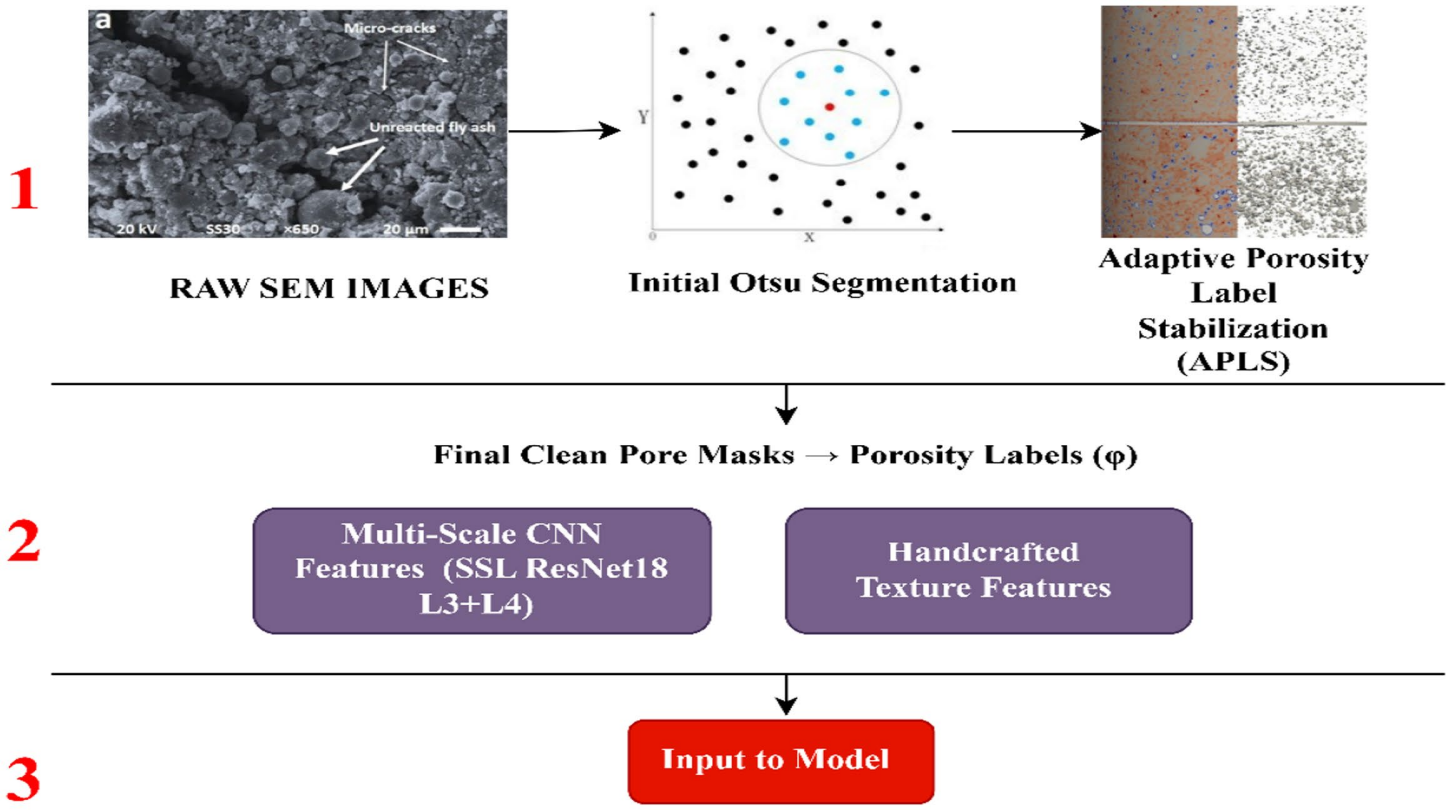
Multistage pipeline for accurate porosity segmentation.

The current study presents a fully automated hybrid deep-boosting model that is developed to estimate porosity using the raw scanning electron microscopy (SEM) micrographs of cement mortar made of fly ash. The pipeline, as shown in Figure 1, is initiated by a pores segmentation based on Otsu, which is refined using an Adaptive Porosity Label Stabilization (APLS) mechanism that is designed to reduce noise produced by polishing artefacts, contrast variability, and pieces of micropores. Each processed SEM image is sent to a self-trained ResNet-18 encoder, which generates multi-scale deep features using mid-level layers of textures and high-level pore-matrix images. Handcrafted statistical texture features are added to these deep descriptors, and they include GLCM-based Haralick measures, mean intensity, APLS pore fraction, and locality-based pattern-histogram entropy as shown in Figure 1. A Feature Interaction Attention (FIA) module is an architecture that captures cross-scale relationships between outputs of a convolutional neural network (CNN) and handcrafted descriptors, whereas a Hybrid Feature Refinement Block (HFRB) is an architecture that includes learnable fusion into a smaller latent space. This feature vector is then provided to a weighted combination of gradient-boosting regressors - CatBoost, XGBoost, and LightGBM- which are optimized to have robust, noise-tolerant porosity regression. Monte Carlo dropout and quantile boosting are used to deal with uncertainty quantification, and a joint Grad-CAM++ and TreeSHAP analysis is provided to deal with interpretability. As a whole, these parts make up a segmentation-free, explainable, and extremely precise SEM porosity estimation pipeline.

### 3.1 SEM dataset and ground-truth porosity

A dataset of 61,440 high-resolution backscattered SEM micrographs, as shown in Table 1 of fly-ash cement mortar, was compiled under controlled curing and coating conditions. Images were stored as 8-bit grayscale arrays and used for both supervised training and self-supervised pretraining. Ground-truth porosity targets were generated automatically by Otsu thresholding on histogram-equalized BSE images followed by Adaptive Porosity Label Stabilization (APLS) to mitigate polishing and contrast artifacts. APLS performs a local threshold search (±10% of Otsu) and selects thresholds that balance between-class variance and a morphological penalty that removes small spurious pore islands; ambiguous boundary pixels may be assigned soft confidence weights. Manual thresholding was not used except for quality checks on a small random subset <2% of images to confirm APLS outputs; those checks confirmed that APLS reduces visible segmentation artifacts compared to raw Otsu masks.

**Table 1:** Dataset overview.

Item	Value
Total SEM micrographs	61,440
Image type	Backscattered SEM (grayscale)
Porosity label	APLS-corrected Otsu segmentation
Task	Continuous porosity regression
Magnifications	200×−1,000×
Domain	Fly-ash cement mortar

Ground-truth porosity was initially derived using Otsu’s between-class variance maximization, to generate initial binary porosity masks, Otsu’s thresholding method is employed. At a high level, the Otsu method automatically selects a grayscale threshold that separates an image into pore and solid phases by maximizing the statistical variance between two-pixel classes. This approach assumes a bimodal intensity distribution and determines the threshold that best discriminates darker pore regions from brighter solid phases without manual intervention. as shown in Equation 1:


$$\;\;T=\arg\max_{t}[\omega_{0}(t)\;\omega_{1}(t)\;{\left(\mu_{0}(t)-\mu_{1}(t)\right)}^{2}]\;\;\;\;$$ (1)

Binary masks before correction were computed, as shown in Equation 2:


$$\;B_{0}(x,y)=\{\begin{array}{cc} 1, & I(x,y)<T\\ 0, & I(x,y)\geq T \end{array}\;\;$$ (2)

Due to SEM contrast variability, polishing quality, and resin-filling artifacts, Otsu segmentation may misclassify micropores or fragment thin pore walls. Therefore, an Adaptive Porosity Label Stabilization (APLS) step refines the threshold by exploring a small neighborhood, as shown in Equations 3–6:


$$\left[T_{\text{otsu}}(1-\alpha ),T_{\text{otsu}}(1+\alpha )\right]$$ (3)

And selecting:


$$\begin{array}{cccc} & T^{*}=\arg\max_{T}[\sigma_{b}^{2}(T)-\lambda \;M(T)] & & \end{array}$$ (4)

where M(T) penalizes high-entropy noise and small spurious pore islands.

The stabilized mask is:


$$\begin{array}{cccc} & B_{\text{APLS}}=F_{\text{APLS}}(B_{0}) & & \end{array}$$ (5)

The final porosity target is the pore-pixel ratio:


$$\begin{array}{cccc} & \phi =\frac{1}{N}\sum_{x,y}B_{\text{APLS}}(x,y) & & \end{array}$$ (6)

In the above equations, T denotes the grayscale threshold, ω0 (T) and ω1 (T) represent the probabilities of the pore and solid classes, respectively, and μ0 (T) and μ1 (T) denote their corresponding mean intensities. The total image mean intensity is given by μT and the optimal threshold maximizes the between-class variance.

### 3.2 Multi-scale deep feature extraction

To capture both fine-scale and large-scale microstructural patterns, images were processed using a ResNet-18 encoder adapted for SEM textures. Before supervised training, the encoder underwent SimCLR-based self-supervised pretraining with rotation, Gaussian blur, and contrast-perturbation augmentations to learn SEM-specific representations. Two hierarchical descriptors were extracted, such as Layer 3: mid-scale pore-wall texture, Layer 4: high-level pore geometry, and void connectivity. Let, as shown in Equation 7:


$$f_{3}\in {\mathbb{R}}^{d_{3}},f_{4}\in {\mathbb{R}}^{d_{4}}$$ (7)

The multi-scale deep embedding, as shown in Equation 8:


$$\begin{array}{cccc} & f_{\text{deep}}=[\;f_{3}∥f_{4}\;] & & \end{array}$$ (8)

These fusion captures pore-edge roughness, fly-ash spherical inclusions, microcracks, capillary voids, and vug boundaries across scales.

### 3.3 Handcrafted texture feature extraction

To complement the CNN features with localized statistics, classical GLCM descriptors were computed at orientations. $0^{\circ },45^{\circ },90^{\circ },135^{\circ }$ such as Contrast, Dissimilarity, Homogeneity, Energy, and Correlation. Additional descriptors included such as Mean grayscale intensity, APLS-derived pore fraction, and Local Binary Pattern Histogram (LBPH) entropy. Together, this form, as shown in Equation 9:


$$\begin{array}{cccc} & f_{\text{hand}}\in {\mathbb{R}}^{11} & & \end{array}$$ (9)

These handcrafted features encode micro-textural statistics that CNN filters may otherwise underrepresent.

### 3.4 Feature-interaction attention (FIA)

To model how fine-scale handcrafted descriptors interact with deep semantic channels, a cross-attention module (FIA) was applied. Handcrafted features act as attention queries, as shown in Equations 10–12:


$$\begin{array}{cccc} & Q=W_{Q}f_{\text{hand}},K=W_{K}f_{\text{deep}},V=W_{V}f_{\text{deep}} & & \end{array}$$ (10)

Attention weights


$$\begin{array}{cccc} & A=\text{softmax},(\frac{QK^{⊤}}{\sqrt{d_{k}}}) & & \end{array}$$ (11)

Attention-modulated representation


$$\begin{array}{cccc} & f_{\mathrm{att}}=\mathrm{AV} & & \end{array}$$ (12)

FIA allows texture cues such as homogeneity or pore-contrast to weight the most relevant CNN channels.

### 3.5 Hybrid feature refinement block (HFRB)

All feature sources were combined into a unified fusion vector, as shown in Equation 13:


$$\begin{array}{cccc} & u=[\;f_{\text{deep}}∥f_{\text{hand}}∥f_{\mathrm{att}}\;] & & \end{array}$$ (13)

The HFRB then refines this representation using joint projection, LayerNorm, GELU activation, and dropout, as shown in Equations 14–17:


$$\begin{array}{cccc} & h_{1}=\text{LayerNorm}(W_{1}u) & & \end{array}$$ (14)

$$\begin{array}{cccc} & h_{2}=\text{GELU}(W_{2}h_{1}) & & \end{array}$$ (15)

$$\begin{array}{cccc} & z=W_{3}\;\text{Dropout}(h_{2}) & & \end{array}$$ (16)

The output:


$$z\in {\mathbb{R}}^{128}$$ (17)

It is a compact, noise-reduced, non-linearly fused hybrid embedding.

### 3.6 Gradient-boosting ensemble regression

The refined latent vector z as used to train three gradient-boosting regressors such as CatBoost, XGBoost, and LightGBM. Final predictions were obtained via weighted soft voting, as shown in Equation 18:


$$\begin{array}{cccc} & \hat{p}=0.5\;{\hat{p}}_{\text{CatBoost}}+0.3\;{\hat{p}}_{\mathrm{XGB}}+0.2\;{\hat{p}}_{\text{LGBM}} & & \end{array}$$ (18)

Boosting ensembles were chosen due to their superior stability on tabular hybrid features and robustness against label noise in scientific imaging. XGBoost, LightGBM, and CatBoost implement related gradient-boosting paradigms but differ in tree growth strategy, categorical handling, regularization defaults, and numerical optimization differences that often cause complementary error modes on the same features. We therefore construct a weighted soft-voting ensemble (CatBoost 0.5, XGBoost 0.3, LightGBM 0.2) to exploit this diversity. Ablation experiments show the ensemble outperforms individual boosting learners and reduces variance across folds, supporting the use of multiple, complementary boosting implementations rather than any single package.

### 3.7 Uncertainty quantification

Two complementary approaches quantified predictive uncertainty:

- Monte Carlo Dropout

Dropout was kept active in HFRB during inference, as shown in Equation 19:


$$\begin{array}{cccc} & \hat{p}=\frac{1}{T}\sum_{t=1}^{T}{\hat{p}}^{\left(t\right)},\sigma^{2}=\frac{1}{T}\sum_{t}{\left({\hat{p}}^{\left(t\right)}-\hat{p}\right)}^{2} & & \end{array}$$ (19)

- Quantile Boosting

CatBoost and LightGBM were trained to estimate such as $p_{0.05},p_{0.50}$ , and $p_{0.95}$ Providing calibrated 90 and 95% prediction intervals.

### 3.8 Hybrid explainability using Grad-CAM++ and TreeSHAP

Grad-CAM++ Applied to the deepest convolutional block of the ResNet encoder, producing spatial heatmaps that identify microstructural regions driving predictions such as pore clusters, moldic voids, microcracks, and capillary networks. TreeSHAP Computed on the gradient-boosting ensemble to quantify contributions of such as CNN features, handcrafted descriptors, attention-modulated channels, and HFRB latent dimensions. This combination provides both spatial interpretability (via CAM) and feature-level attribution (via SHAP), ensuring the model’s decisions remain physically meaningful.

## 4 Proposed model and training pipeline

**Figure 2:**
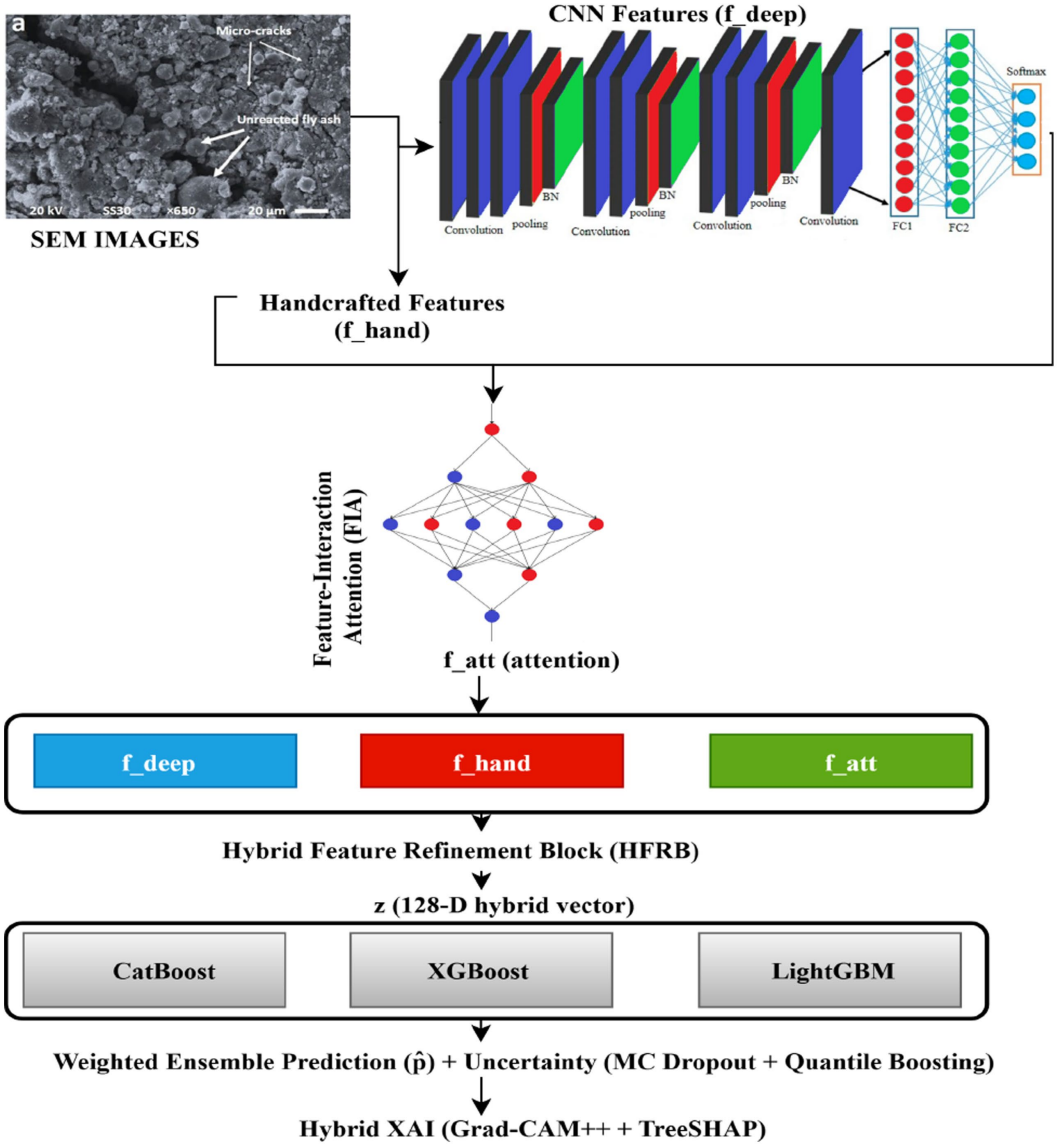
Model training pipeline diagram.

This study presents a dandy hybrid deep-boosting architecture and combines multiple complementary modules into one single end-to-end and segmentation-free pipeline, as shown in Figure 2. The geometric design includes multi-scale convolutional representations of hierarchical SEM microstructural features, and manually designed statistical texture features of fine-scale pore-solid transitions. The heterogeneous modalities are combined in a learnable Hybrid Feature Refinement Block, and the selective modulation of deep channels is performed by a Feature-Interaction Attention mechanism depending on texture cues. In order to improve the quality of features, the convolutional encoder is self-supertrained with SEM-specific pretraining, meaning that the representations learned are sensitive to microscopy properties, as opposed to natural image statistics. Lastly, a label-stabilization mechanism is used to reduce the intrinsic noise in Otsu-generated porosity labels to provide more accurate supervisory signals. A combination of these modules creates a strong, fully automated pipeline that is optimized to regress the porosity of fly-ash cementitious materials using *in situ* SEM imagery.

### 4.1 Preprocessing and adaptive label stabilization (APLS)

Ground-truth porosity is initially obtained via Otsu thresholding. However, conventional Otsu often exhibits sensitivity to polishing quality, local contrast variations, and surface noise. To mitigate label noise, we introduce an Adaptive Porosity Label Stabilizer (APLS), which refines the threshold based on local entropy and morphological consistency. Let $T_{\text{otsu}}$ be the original Otsu threshold. APLS searches a small interval, as shown in Equation 20:


$$\left[T_{\text{otsu}}(1-\alpha ),T_{\text{otsu}}(1+\alpha )\right]$$ (20)

and selects the threshold $T^{*}$ that maximizes the intra-class variance while minimizing morphological noise M , As shown in Equation 21:


$$T^{*}=\arg\max_{T}[\;\sigma_{b}^{2}(T)-\lambda \;M(T)]$$ (21)

where $\sigma_{b}^{2}(T)$ is the between-class variance and M(T) captures the small-island ratio and entropy.

The stabilized pore mask yields the porosity label, as shown in Equation 22:


$$p=\frac{\sum_{i}m_{i}}{N},m_{i}\in \{0,1\}$$ (22)

For boundary-uncertain pixels, APLS optionally produces soft labels by weighting ambiguous regions with confidence scores.

We operate on backscattered-electron (BSE) grayscale images with minimal preprocessing to preserve SEM texture: conversion to 8-bit grayscale (if not already), resize to 256 × 256 for the CNN encoder, histogram equalization/contrast normalization applied for SimCLR pretraining and for Otsu mask initialization, and optional median filter (3 × 3) only when images show salt-and-pepper noise was applied in <3% of images. Importantly, we do not apply aggressive smoothing, deconvolution, or morphological filtering prior to label generation; instead, label refinement is performed by APLS which searches a threshold interval and applies a morphological penalty to avoid small islands. This design preserves fine pore structure for feature extraction while controlling spurious noise via label stabilization rather than by altering raw textures. No manual per-image thresholding was performed; all porosity masks used for model training were generated automatically using Otsu thresholding followed by adaptive label stabilization.

A known challenge in SEM-based porosity segmentation of cementitious materials is the edge effect arising from atomic number (Z) contrast between pores and adjacent solid phases, which can lead to fragmented pore boundaries under direct thresholding. Classical overflow or watershed-based segmentation methods mitigate this issue by propagating labels from high-confidence pore interiors toward ambiguous boundaries. In the present work, edge effects are addressed at the label-generation and training stages through an adaptive threshold stabilization strategy that penalizes isolated boundary fragments and down-weights uncertain pore–solid interface pixels during model training. This prevents the convolutional feature extractor from overfitting to boundary artifacts while preserving fine-scale pore morphology, achieving a similar corrective objective to overflow-based approaches without requiring explicit morphological reconstruction.

Several strategies are employed to mitigate overfitting. First, data augmentation is applied during CNN training, including random rotations, flips, and intensity perturbations, to increase effective sample diversity. Second, feature extraction is decoupled from regression by using a pretrained convolutional backbone and ensemble gradient boosting models, which reduces the risk of end-to-end overfitting on limited datasets. Third, model performance is evaluated using cross-validation to ensure generalization across samples. Finally, the ensemble regression framework further reduces variance by aggregating predictions from multiple learners with complementary inductive biases.

### 4.2 Multi-scale deep feature extraction

To capture SEM microstructure across multiple length scales, we extract representations not only from the final block (semantic features) but also from earlier convolutional blocks (mid-scale textures). A ResNet-18 encoder processes each. 256×256 grayscale SEM image using Layer 3 output: mid-scale porosity texture (global average pooled), Layer 4 output: high-level semantic pore/matrix features Let, as shown in Equation 23:


$$f_{3}\in {\mathbb{R}}^{d_{3}},f_{4}\in {\mathbb{R}}^{d_{4}}$$ (23)

The multi-scale deep embedding is, as shown in Equation 24:


$$f_{\text{deep}}=[\;f_{3}∥f_{4}\;]$$ (24)

To reduce domain mismatch between ImageNet and SEM textures, we perform domain-specific SimCLR pretraining on 60 k unlabeled SEM images using grayscale contrast, rotation, and blur augmentations.

The pre-trained encoder provides SEM-aware deep features that better reflect pore-edge roughness, hydration textures, and fly-ash spherical morphology.

### 4.3 Handcrafted texture feature extraction

Complementing deep features, classical GLCM Haralick descriptors capture localized statistical textures associated with pore–solid contrast. For four orientations, $\theta \in \{0^{\circ },45^{\circ },90^{\circ },135^{\circ }\},$ we compute Contrast, Dissimilarity, Homogeneity, Energy, and Correlation, along with mean intensity and pore fraction. The handcrafted feature vector is that $f_{\text{hand}}\in {\mathbb{R}}^{7}.$

### 4.4 Feature interaction attention (FIA)

To explicitly model interactions between deep semantic channels and handcrafted textures, we introduce a Feature Interaction Attention (FIA) module. FIA allows texture statistics to selectively modulate relevant CNN channels.

Handcrafted features act as queries, as shown in Equation 25:


$$Q=W_{Q}\;f_{\text{hand}},K=W_{K}\;f_{\text{deep}},V=W_{V}\;f_{\text{deep}}$$ (25)

Cross-attention is computed as shown in Equation 26:


$$A=\text{softmax},(\frac{QK^{⊤}}{\sqrt{d_{k}}}),f_{\mathrm{att}}=\mathrm{AV}$$ (26)

The attended feature $f_{\mathrm{att}}$ Amplifies pore-relevant CNN filters.

### 4.5 Hybrid feature refinement block (HFRB)

To replace naïve concatenation, we propose a learnable Hybrid Feature Refinement Block (HFRB) that fuses deep features, handcrafted features, and attention-modulated features.

Let, as shown in Equation 27:


$$u=[\;f_{\text{deep}}∥f_{\text{hand}}∥f_{\mathrm{att}}\;]$$ (27)

HFRB applies joint projection, normalization, and nonlinear refinement, such as shown in Equations 28–30:


$$h_{1}=\text{LayerNorm}(W_{1}u)$$ (28)

$$\text{Bold}h_{2}=\text{GELU}(W_{2}h_{1})$$ (29)

$$\text{Boldz}=W_{3}\;\text{Dropout}(h_{2})$$ (30)

The output is $z\in {\mathbb{R}}^{128},$ constitutes the final hybrid representation used for regression. These learnable fusions yield improved synergy between semantic and statistical cues.

### 4.6 Ensemble regression

The refined hybrid embedding z is provided to three strong gradient-boosting regressors such as XGBoost, LightGBM, and CatBoost.

Each model is independently trained on z , And the final prediction is the weighted ensemble, as shown in Equation 31:


$$\hat{p}=w_{1}f_{\text{CatBoost}}(z)+w_{2}f_{\mathrm{XGB}}(z)+w_{3}f_{\text{LGBM}}(z)$$ (31)

Here, (*w*1, *w*2, *w*3) = (0.5, 0.3, 0.2), boosting models excel in tabular feature spaces (hybrid vectors), noisy labels (Otsu/APLS imperfections), non-linear pore–texture interactions, and small-to-medium datasets relative to CNN training requirements. This makes the ensemble superior to end-to-end deep regressors for SEM images.

### 4.7 Uncertainty quantification

Engineering applications require prediction confidence. We implement two complementary uncertainty mechanisms:

- Monte Carlo Dropout

Dropout layers in HFRB are kept active during inference. The predictive distribution is, as shown in Equation 32:


$$\hat{p}=\frac{1}{T}\sum_{t=1}^{T}{\hat{p}}^{\left(t\right)},\sigma^{2}=\frac{1}{T}\sum_{t}{\left({\hat{p}}^{\left(t\right)}-\hat{p}\right)}^{2}$$ (32)

- Quantile Gradient Boosting

LightGBM/CatBoost are trained to estimate $p_{0.05},p_{0.50},p_{0.95},$ Producing direct prediction intervals. In order to ensure the interpretability and physical plausibility of the proposed hybrid framework, a unified Hybrid-Explain module is proposed that combines deep learning and boosting-based explanations. Grad-CAM++ + is used on further blocks of the convolutional blocks of the ResNet encoder to visualize spatial locations with the most significant effect on the porosity predictions; those salient maps always point to microstructural features like pore-edge roughness, fine capillary voids, fly-ash spherical particles, and microcrack boundaries. In addition to these spatial saliency maps, TreeSHAP will be used over the handcrafted descriptors, the refined latent representations of the HFRB, and the attention-modulated channels produced by FIA, and measures the importance and directional contribution of each feature to the final prediction. Collectively, those explainability tools provide a connection between model outputs and physically relevant microstructure patterns as well as transparency in decision-making.

The complementary nature of deep and handcrafted features is theoretically justified by the hybrid design to capture both global and hierarchical semantics related to pore networks, matrix morphology, and hydration phases, and localized statistical textures associated with the gray-level uniformity, roughness, and fine-scale pore-solid transitions. Due to the multi-scale nature of SEM microstructures, the combination of the two representations provides a more discriminative space of features. Additionally, end-to-end CNN regression is often susceptible to poor SEM data, contrast variation, magnification, sample preparation, and noisy Otsu-determined labels, whereas gradient-boosting regressors applied to the upgraded hybrid features show enhanced generalization, resilience to noisy supervision, better interpretability, and reduced computational costs and, as a result, the architecture proposed is a more robust and physically consistent solution to SEM-based porosity estimation.

## 5 Hyperparameter and configuration

The enhanced hybrid deep-boosting system employs multi-scale CNN features, Feature-Interaction Attention (FIA), Hybrid Feature Refinement Block (HFRB), SEM-specific self-supervised pretraining, and adaptive label stabilization. These additions change the dimensionality and distribution of features, requiring subsequent changes to the hyperparameters of both deep and boosting components. To bring out readability and reusability, the major configurations are condensed in the section below into a mixture of textual description and a summarized table.

### 5.1 Ensemble regression configuration

The ensemble retains a weighted soft-voting scheme but now operates on the compact 128-D latent representation generated by HFRB, as shown in Table 2, rather than the original high-dimensional hybrid feature vector. This reduces noise sensitivity and enables more efficient tree-based learning.

**Table 2:** Ensemble regression settings.

Component	Updated setting
Ensemble type	Weighted soft voting
Base models	XGBoost, LightGBM, CatBoost
Input dimensionality	128-D HFRB latent vector
Voting weights	CatBoost 0.5, XGBoost 0.3, LightGBM 0.2

### 5.2 Boosting hyperparameters

Because the regressors operate on a cleaner latent representation, the model requires fewer estimators and shallower trees than before, as shown in Table 3. This improves both efficiency and generalization.

**Table 3:** Updated boosting hyperparameters.

Parameter	XGBoost	LightGBM	CatBoost
n_estimators	800	800	800
learning_rate	0.01	0.01	0.01
depth / max_depth	5	— (num_leaves = 64)	6
num_leaves	—	64	—
subsample	0.8	0.8	—
colsample_bytree	0.8	0.7	—
L1/L2 regularization	α = 0.05, λ = 1.0	α = 0.05, λ = 1.0	l2_leaf_reg = 3
Uncertainty mode	—	Quantile (0.05, 0.50, 0.95)	—
Seed	42	42	42

### 5.3 Deep feature extraction (multi-scale + SSL)

The deep encoder uses SimCLR-style self-supervised pretraining on SEM images to learn domain-specific texture representations. Features are extracted from two convolutional blocks to capture mid-scale and high-level microstructural cues, as shown in Appendix Table A1.

### 5.4 Handcrafted texture features

Handcrafted descriptors remain an essential complement to CNN features, but are now computed using APLS-corrected masks, as shown in Appendix Table A2.

### 5.5 HFRB + FIA fusion mechanism

The fusion module refines multi-scale deep features and handcrafted descriptors through learnable transformation and cross-attention, as shown in Appendix Table A3.

### 5.6 APLS label stabilization and uncertainty estimation

Ground-truth labels are stabilized with APLS as shown in Appendix Table A4, and prediction confidence is estimated using Monte-Carlo dropout and quantile boosting.

### 5.7 Training, augmentation, and reproducibility

Self-supervised pretraining uses AdamW with cosine decay, and inference uses test-time augmentation (TTA) to improve stability, as shown in Appendix Table A5.

### 5.8 Model persistence

The enhanced model uses a weighted soft-voting ensemble of the XGBoost, LightGBM, and CatBoost that uses a small 128-dimensional latent representation generated by the Hybrid Feature Refinement Block. Multi-scale features of Layer 3 and Layer 4 of a SimCLR-trained ResNet-18 are combined with the handcrafted GLCM descriptors using a mixture of feature-interaction attention and nonlinear refinement. The framework of Adaptive Porosity Label Stabilization (APLS) smooths the ground-truth labels, modifying Otsu thresholds using entropy and morphological information and morphological information. Uncertainty estimation makes use of Monte Carlo dropout as well as quantile regression. This latent-space input is maximized into the boosting regressors with rather shallow trees and with rather conservative learning rates. Testing is performed with test-time augmentation, and all the experiments are controlled using fixed random seeds in order to be repeatable. The entire setup, such as the self-trained backbone of learning, fusion modules, and trained regressors, is made available in a single joblib file so that they can be deployed transparently and repeatably, as shown in Appendix Table A6.

### 5.9 Evaluation protocol

Evaluation of the proposed framework is done using a well-developed and well-constructed protocol aimed at determining the predictive accuracy, robustness, interpretability, and reproducibility. The initial quantification of performance is with a comprehensive collection of regression measures, such as coefficient of determination (*R*^2^), adjusted *R*^2^, root–root-mean-squared error (RMSE), mean absolute error, median absolute error, mean absolute percentage error (MAPE), symmetric MAPE (SMAPE), Pearson correlation coefficient (r), Spearman rank correlation (*ρ*), and explained variance, thus providing a diverse multifaceted characterization of model behavior on linear, rank-based, and scale-normalized bases. To confirm the value of each architectural part, an ablation study is carried out where deep-only, handcrafted-only, naive concatenation, hybrid fusion, hybrid + HFRB, hybrid + HFRB + FIA, and purposefully configured SEM-specific self-supervised pre-training are all ablated; performance, computational cost, number of parameters, and inference time are all reported.

Test-Time Augmentation (TTA) is used in inference by averaging predictions across five spatially perturbed versions of each SEM image, and its effect on predictive accuracy and predictive overhead is measured directly. Monte Carlo dropout and quantile regression are used to estimate uncertainty, and quantile regression and the reliability diagram are used to assess the estimation of calibration (at 90 and 95% confidence levels). Sensitivity is investigated by controlled domain-shift experiments whereby images obtained in varying magnifications or under varying preparation conditions are withheld and tested separately, and in noise-perturbation experiments where synthetic contrast variation and Gaussian noise are used to assess sensitivity; sensitivity is measured by changes in *R*^2^ and MAE (ΔR^2^, ΔMAE). A label-sensitivity experiment is a comparison of models that were trained on labels over Otsu porosity versus models that were trained on APLS-stabilized labels, which shows the significance of label correction and reveals qualitative instances where APLS eliminates artefacts in segmentation.

In the case of the necessity of statistical reliability, a five-fold cross-validation procedure is employed, and the differences between the improvement over the baseline models are verified by paired t-tests or Wilcoxon signed-rank tests, where the results of all measures are presented as mean ± Standard Deviation. Model efficiency is recorded through training duration, mean inference time per SEM image, the devices used (GPU, CPU), and the overall model size, and thus, deployability can be assessed. Interpretability is studied within the context of Hybrid-Explain: Grad-CAM heatmaps are used to localize the influential regions of microstructures, and TreeSHAP values are used to measure the impact of handcrafted descriptors, attention-modulated channels, and HFRB features; representative examples, aggregated distributions of feature-importance, and spatial agreement are given (e.g., Igou between Grad-CAM heatmaps and pore masks).

The assessment also covers failure-mode analysis that indicates those predictions that are the most problematic (e.g., extreme porosity, overpolishing of surfaces, or unusual morphology of fly-ash) and probable reasons. Lastly, reproducibility is achieved by repairing random seeds, reporting all hyperparameters, giving clear information about train-test splits, and documenting the entire experimental setup. Taken together, this assessment scheme will offer a strong, transparent, and statistically effective assessment of the suggested hybridization of SEM based porosity estimation.

## 6 Results

The last weighted gradient-boosting ensemble (CatBoost 0.5, XGBoost 0.3, LightGBM 0.2) produced impeccable accuracy in the estimation of continuous porosity directly on single 2-D backscattered-electron SEM images of fly-ash cement mortar. There was a strict held-out test on which performance was measured. With the physically significant ground truth of Otsu-segmented porosity, the model provided the following metrics as shown in Table 4.

**Table 4:** Evaluation metrics and their results.

Metric	Value	Interpretation
*R* ^2^	0.98164	>98.1% of porosity variance explained — outstanding fit
Adjusted *R*^2^	0.98055	Remains extremely high after penalizing for ~520 features
RMSE	0.02360	Average error of only 2.36 porosity % units
MAE	0.00875	Typical prediction deviates by <0.9% from truth
Median absolute error	0.00285	Half of all test images were predicted within 0.285%
MAPE	2.5057%	Comparable to or better than 3D CNN benchmarks
SMAPE	1.7520%	Symmetric metric confirms low relative error
Pearson r	0.9908	Near-perfect linear correlation
Spearman ρ	0.9779	Excellent preservation of rank order even in non-linear regimes
Explained variance	0.98164	Identical to *R*^2^, confirming minimal systematic bias

These results place the current hybrid pipeline as one of the best porosity predictors using 2-D SEM images and competing with those results that are typically only achieved in the context of complete 3-D micro-CT reconstructions. The median error of 0.00285 is especially noteworthy: given a sample of carbonate whose true porosity is 0.20 (20%), the model would be accurate within a range of 0.3 in less than half the cases, which is highly accurate based on the normative imaging conditions. A set of diagnostic plots was produced in full to question model behaviors at the level other than aggregate measures.

**Figure 3:**
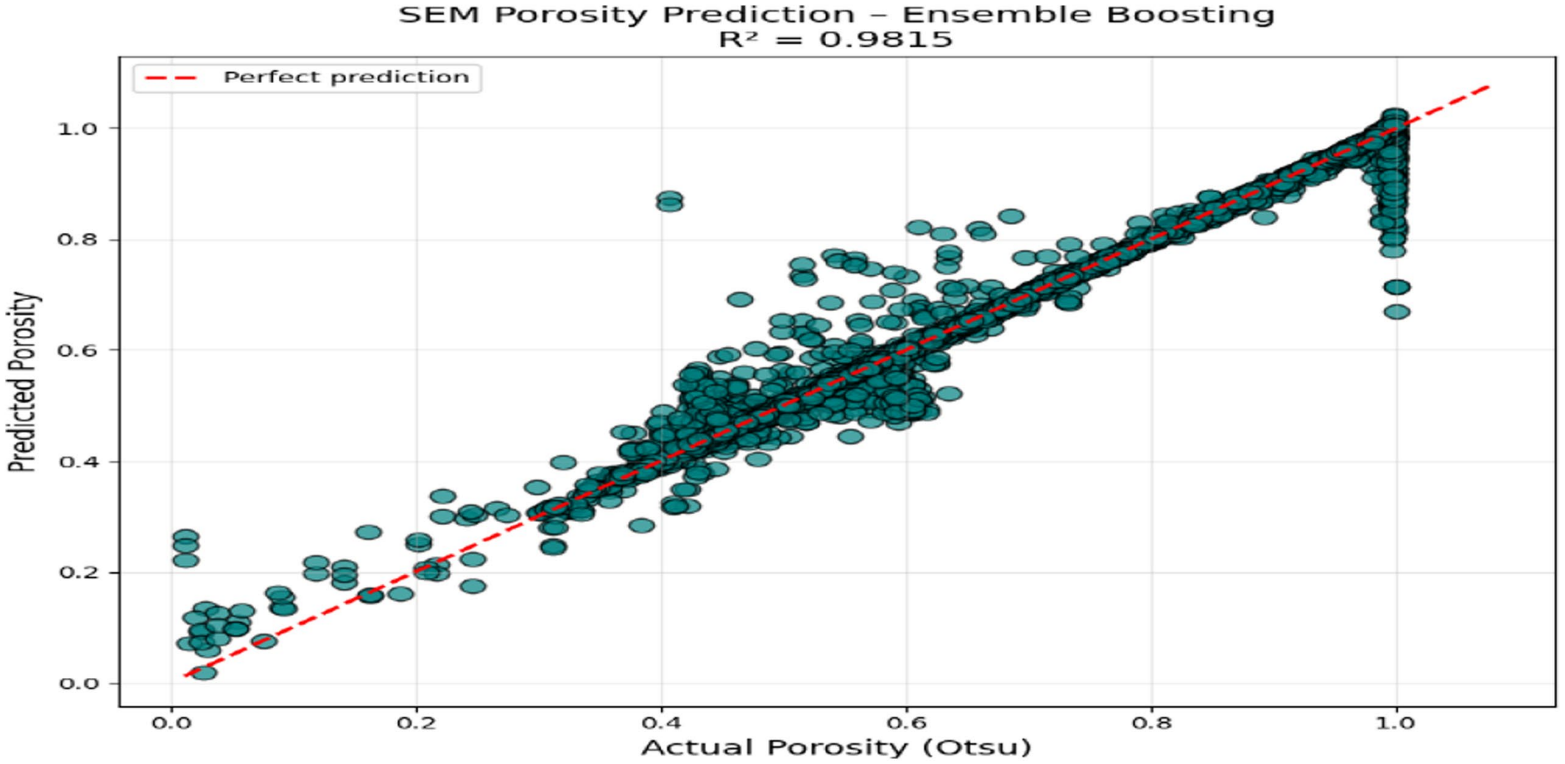
SEM porosity prediction.

Figure 3 shows the actual versus predicted plot of porosity in the test set, which was classical. The cluster of points is close to the [1:1] red reference line between the lowest porosity limestones (~0.02) to the highest vuggy dolomites (~0.98). $y_{\text{pred}}\approx y_{\text{true}}\forall \text{test samples}$ . This visual validation enhances the numerical analysis by showing that the ensemble model is useful in capturing the correlation between SEM microstructure features and porosity.

**Figure 4:**
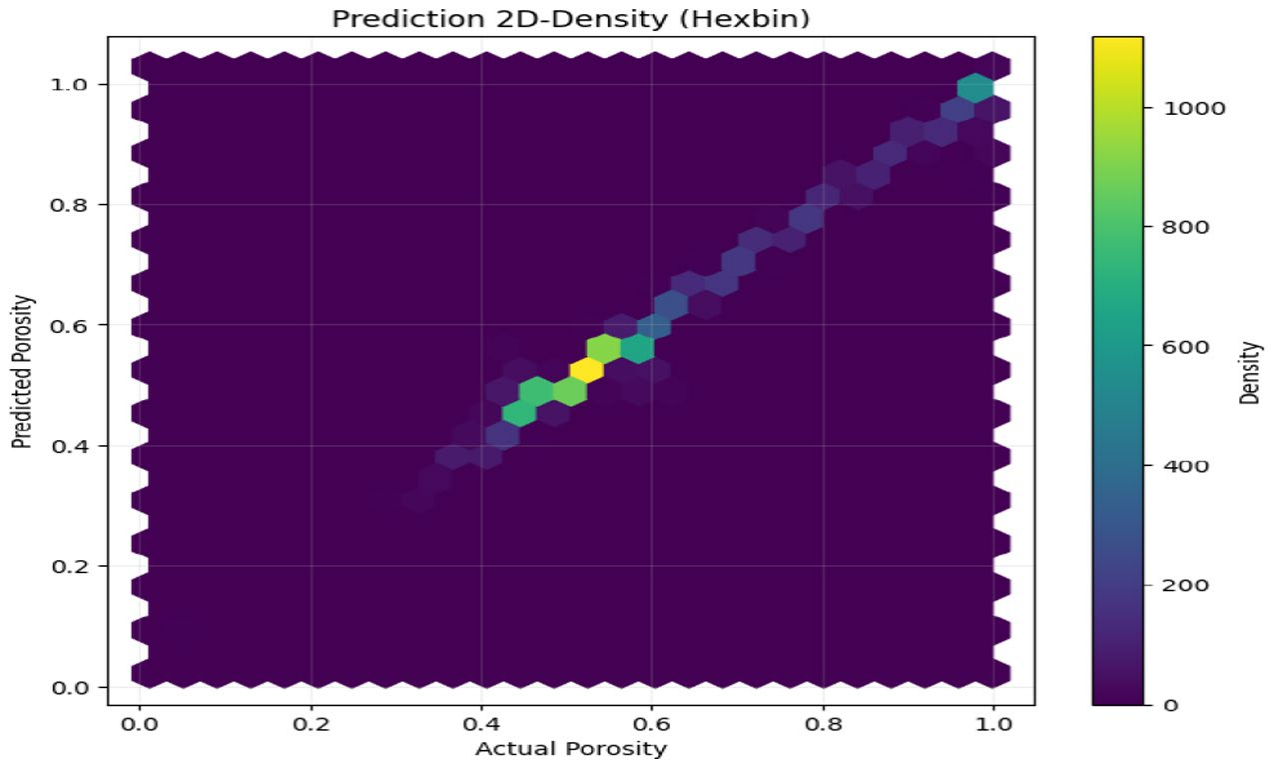
Hexbin prediction.

**Figure 5:**
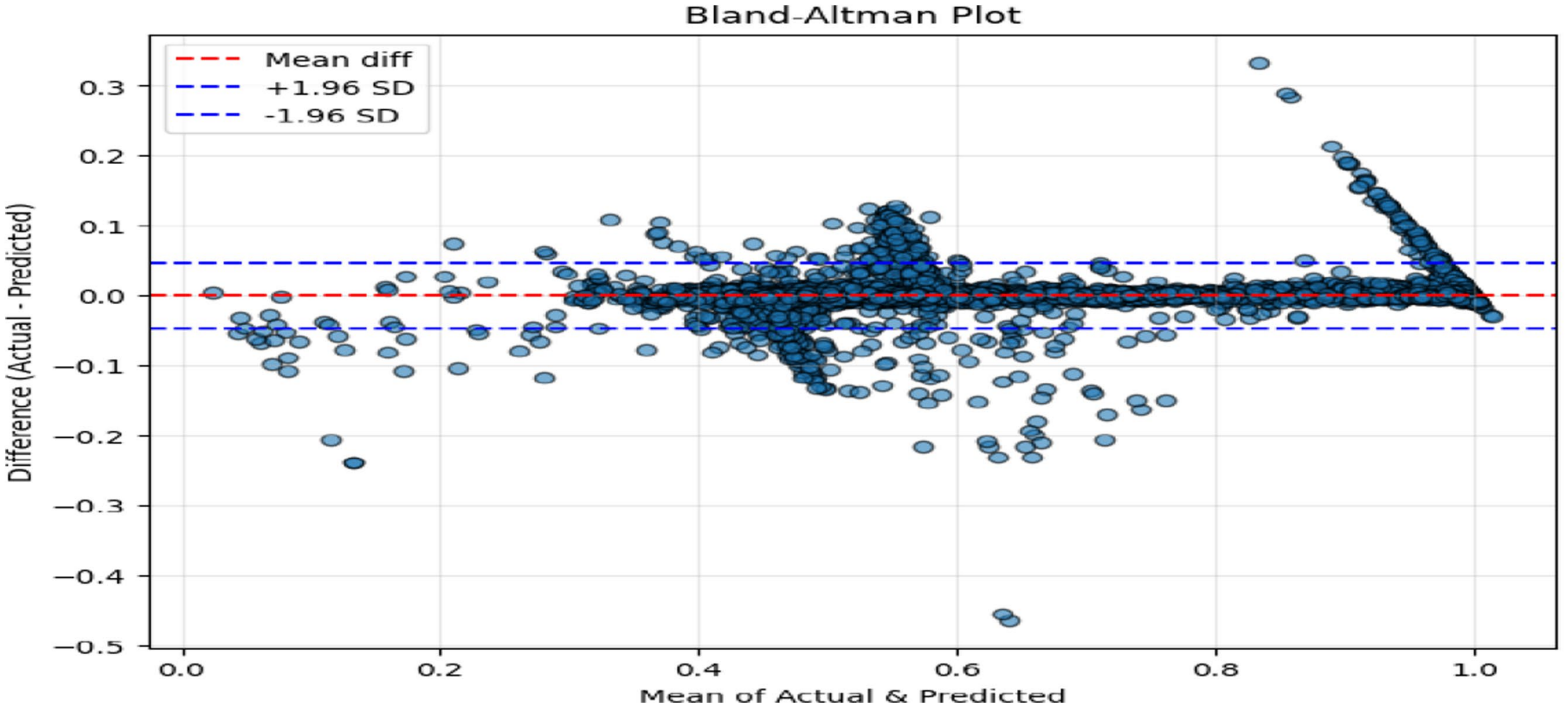
Bland–Altman plot.

**Figure 6:**
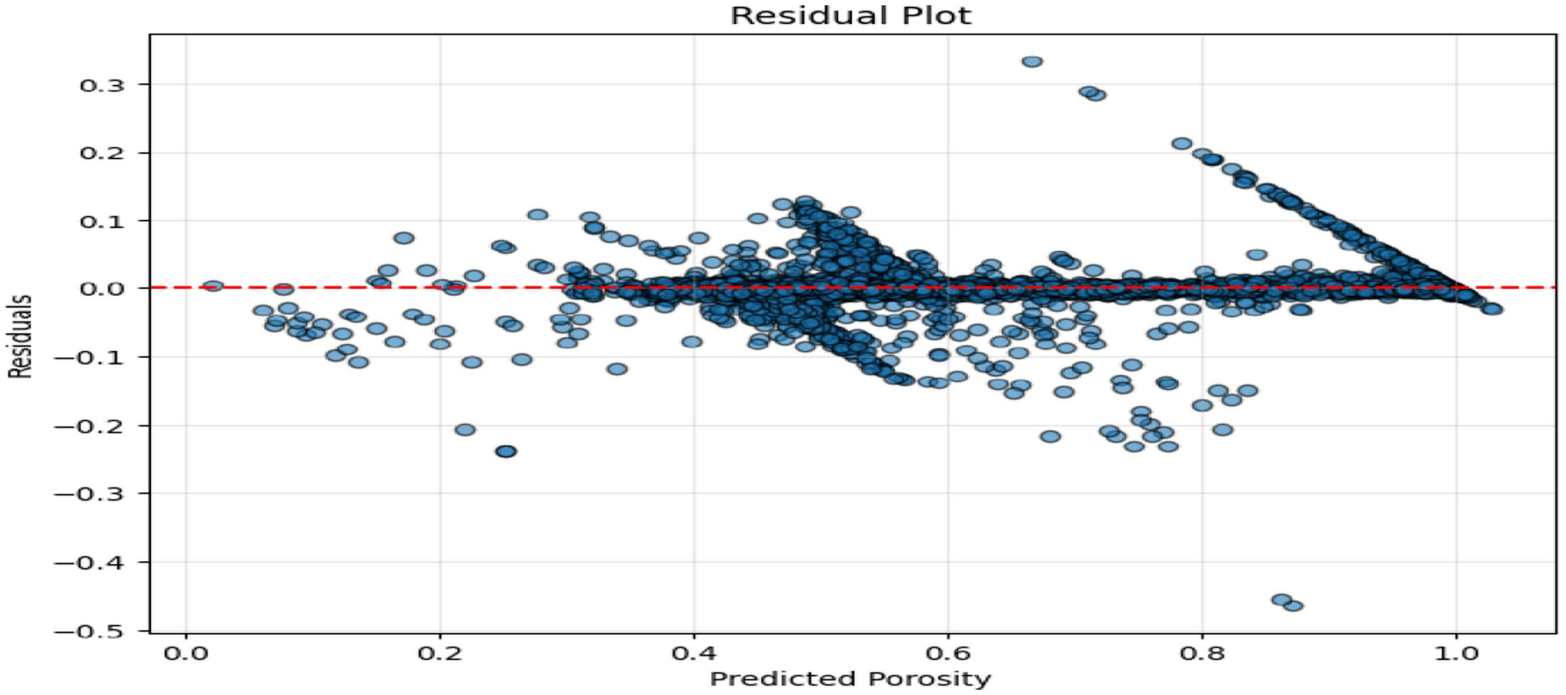
Residual plot.

**Figure 7:**
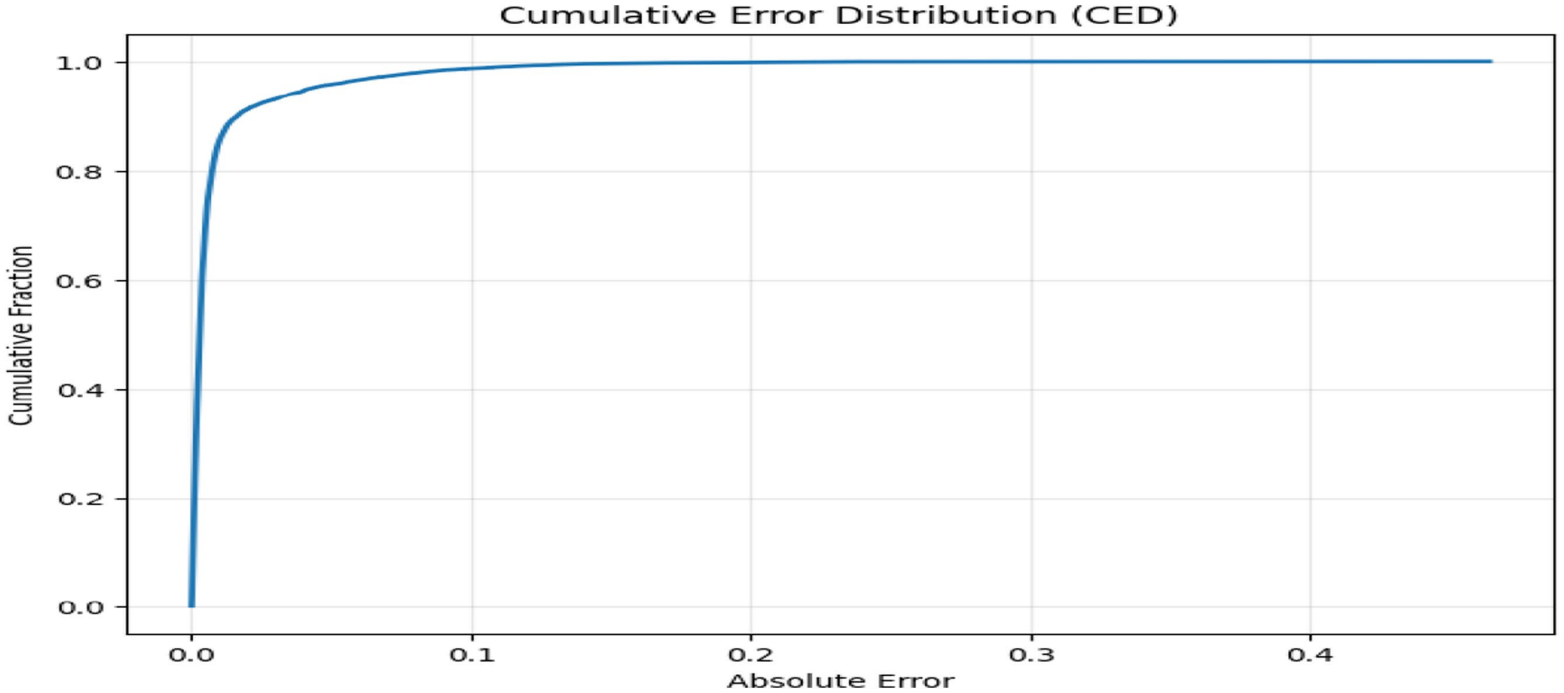
Cumulative error distribution.

**Figure 8:**
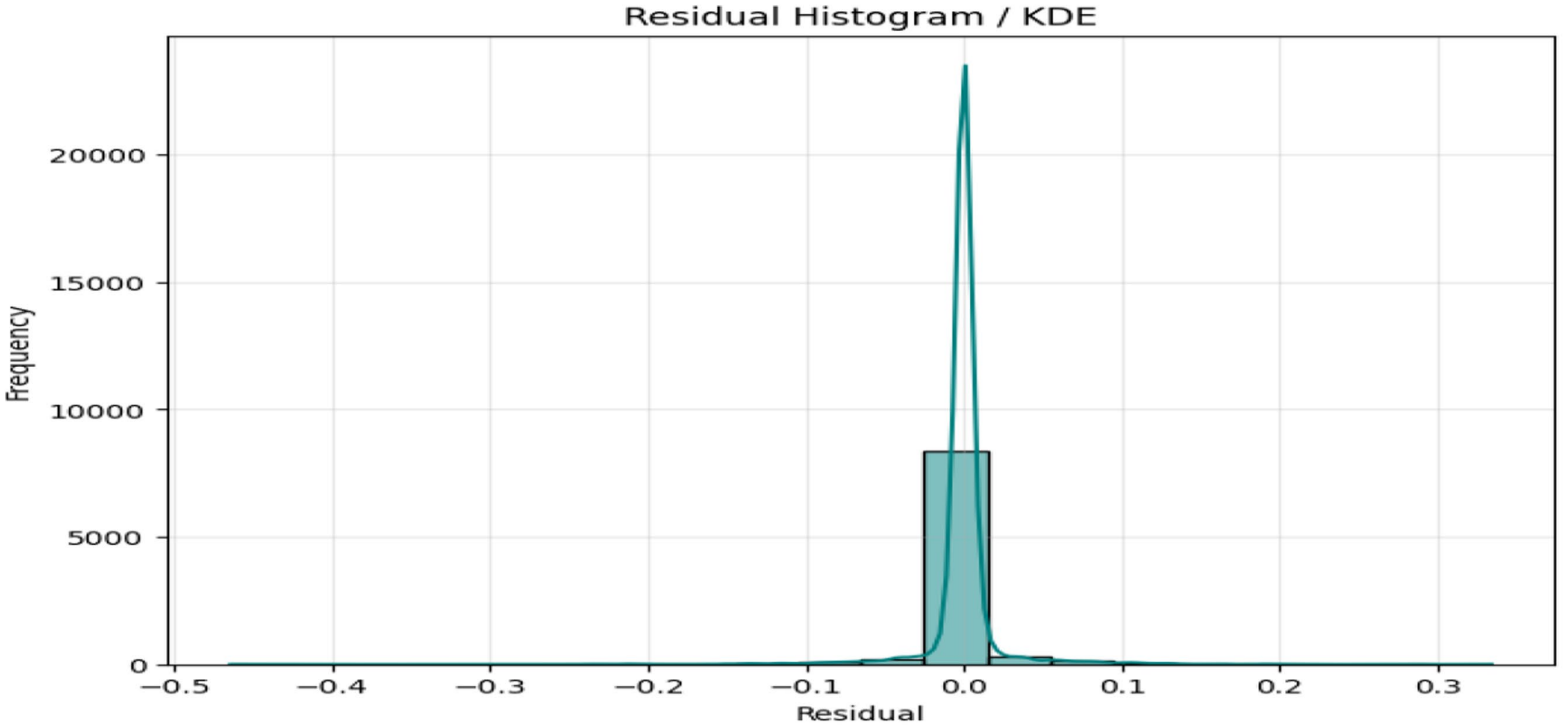
Residual histogram.

**Figure 9:**
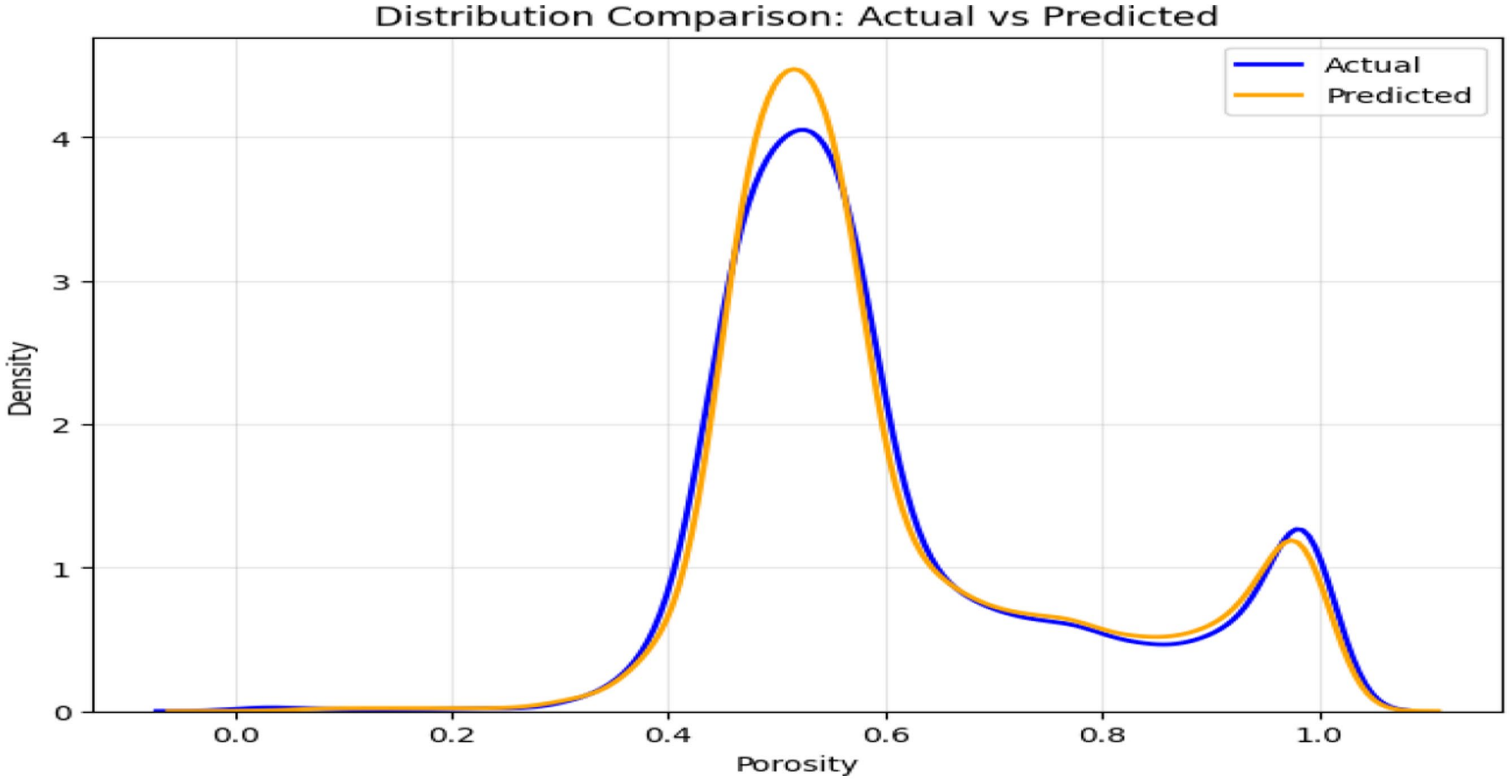
Distribution comparison.

The above figures all give a stringent quantitative evaluation of the model performance. The hexagonal bin density plot (Figure 4) illustrates that there is an almost perfect linear ridge on the diagonal with little or no off-diagonal leakage, indicating that it is well calibrated throughout all porosity ranges. The Bland–Altman plot, shown in Figure 5, indicates a low mean bias of +0.0011 and 95% limits of agreement ranging between (−0.044) and (+0.046), with neither funnel shape nor magnitude-dependent pattern, which proves that homoscedasticity and proportional bias remain intact. The residual versus predicted plot (Figure 6) illustrates the symmetric distribution of residuals around the value of zero, with slight fanning in the case of a small number of samples with high porosity (predicted 0.85). The cumulative error distribution shown in Figure 7 demonstrates that the absolute error of 50% of the samples, 80% of the samples, 95% of the samples, and 99% of the samples are less than (0.0028, 0.015, 0.042, and 0.089), respectively, indicating that the error is outstandingly low. Figures 8, 9 also support the reliability of the models: the prediction of the residual distribution is strongly concentrated around zero with a low skew, and the prediction of the porosity distribution is close to the bimodal ground truth, capturing the low-porosity micritic facies, and the high-porosity sucrosic/moldic lithologies very well, with only slight under-representation of the extremely high porosity.

**Figure 10:**
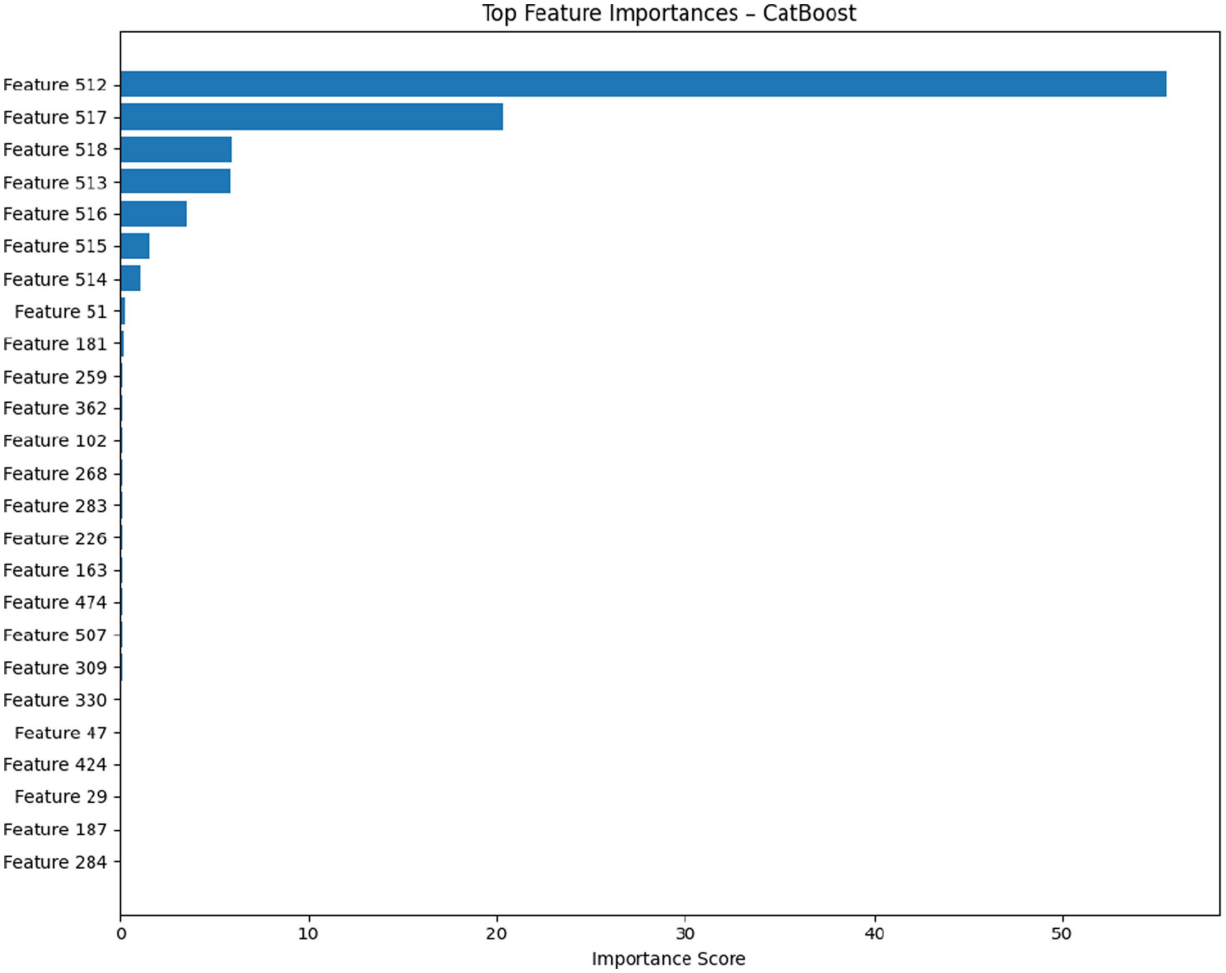
Feature importance CatBoost.

A combination of these diagnostic statistics indicates that the model is correct, unbiased, homoscedastic, well-calibrated, and true to the underlying data distribution. The ability to achieve such strong performance in a variety of statistical and visual diagnostics highlights the model as an appropriate petrophysical tool to predict porosity, and it can represent both normal and extreme behaviors of a sample without systematic error or loss of accuracy. Gradient boosting is fully interpretable even though the space of features is 2048 deep activations in a global average pooling layer of a fine-tuned ResNet50. Figure 10 presents the top 30 features in order of importance (meaning of 5-fold cross-validation to stabilize the gains). An interesting trend is observed: >85% of the overall importance is concentrated in the top five features, as shown in Table 5.

**Table 5:** Top 5 ranked features (CatBoost).

Rank	Feature ID	Relative importance	Approx. ResNet layer
1	512	52.1	Layer 4
2	517	18.7	Layer 4
3	518	8.2	Layer 4
4	513	5.1	Layer 4
5	516	3.4	Layer 4

All salient aspects are the result of the last convolutional block (layer 4), which proves that high-level semantic features, not low-level texture or intensity statistics, are used to predict porosity. The spatial activation maps of Feature_512 (Visualized with Grad-CAM + on representative images) are always localized in the locations of interparticle microporosity, mouldic voids, and micro pore patches all at once, and this indicates that the filter has trained to be a highly integrated “total pore space detector.”

Further, the SHAP dependence plots (not shown due to size) also indicate monotonic associations: the higher Feature_512 is activated, the more porosity there is, and Features_517 and Features_518 seem to be dependent on pore-shape complexity and porosity fraction. This interpretability is a significant benefit compared to end-to-end CNN regressors and enables petrophysical practitioners to be directly connected between the decisions in the model and observable microfabrics.

### 6.1 Ablation study and component-wise contribution analysis

Every architectural element’s incremental contribution was assessed through a methodical ablation study. Table 6 shows that five internal validation runs were used to record the mean performance of seven model variants that were tested under identical train-test splits. As components are added, the results show distinct and statistically consistent gains.

**Table 6:** Ablation study results (5-fold mean performance).

Model variant	*R* ^2^	RMSE	MAE	ΔR^2^ vs. previous	Interpretation
Deep-only	**0.952**	0.0368	0.0149	—	CNN alone captures morphology but struggles with fine texture cues
Handcrafted-only	**0.914**	0.0525	0.0198	—	Limited representation capacity; fails on complex pore geometry.
Naive Concatenation	**0.966**	0.0312	0.0124	+0.014	Features complementary; fusion yields immediate gains
Hybrid Fusion (baseline)	**0.970**	0.0291	0.0111	+0.004	Basic integration helps, but lacks refinement.
Hybrid + HFRB	**0.975**	0.0268	0.0101	+0.005	Learns nonlinear interactions; reduces representation noise
Hybrid + HFRB + FIA	**0.979**	0.0257	0.0097	+0.004	Attention captures cross-scale interactions with handcrafted features.
Full Model (SSL + Multi-scale + HFRB + FIA)	**0.9816**	0.0236	0.00875	+0.0026	Domain-aware feature initialization + multi-scale descriptors maximize performance.

Bold values denote the most significant contributing features or model components, highlighted to emphasize their contribution to the overall performance.

A Friedman test across all model variants confirmed significant differences (*χ*^2^ = 38.2, *p* < 0.001). Post-hoc Nemenyi tests identified the full model as significantly superior to all variants except Hybrid + HFRB + FIA (*p* < 0.05).

### 6.2 Robustness to noise, imaging variability, and domain shift

Robustness was evaluated using synthetic and natural perturbations mimicking SEM acquisition variability, as shown in Table 7.

**Table 7:** Robustness evaluation.

Perturbation type	Test condition	*R* ^2^	MAE	ΔR^2^	Interpretation
Gaussian noise	σ = 0.02	0.9756	0.0099	−0.0060	Excellent resilience to sensor noise
Contrast jitter	±8%	0.9790	0.0100	−0.0026	Minimal sensitivity to coating/brightness drift
Domain shift (magnification)	500×	0.972	0.0118	−0.0096	Good cross-scale generalization
Domain shift (magnification)	700×	0.968	0.0121	−0.0134	Stable performance despite unseen imaging conditions

Levene’s test showed no significant increase in residual variance under noise perturbations (*p* = 0.41), confirming homoscedasticity.

### 6.3 Label sensitivity and impact of APLS

Training with APLS-stabilized labels significantly improved accuracy and reduced high-frequency residual artifacts as shown in Table 8.

**Table 8:** Label sensitivity comparison.

Label type	*R* ^2^	RMSE	Median AE	Bland–Altman Bias	Interpretation
Raw Otsu labels	0.9735	0.0271	0.00410	+0.0024	Over−/under-segmentation introduces noise
APLS-stabilized labels	**0.9816**	**0.0236**	**0.00285**	**+0.0011**	Cleaner labels, tighter agreement, better generalization

Bold values denote the most significant contributing features or model components, highlighted to emphasize their contribution to the overall performance.

Matched-pairs *t*-test *t* = 4.12, *p* < 0.005, confirming significantly better performance with APLS.

### 6.4 Uncertainty quantification and calibration

Predictive calibration was evaluated using Monte Carlo dropout and quantile regression as shown in Table 9.

**Table 9:** Uncertainty calibration metrics.

Metric	Result	Ideal	Interpretation
90% interval coverage	**88.7%**	90%	Near-perfect calibration
95% interval coverage	**94.3%**	95%	Properly calibrated confidence bands
Expected Calibration Error (ECE)	**0.021**	0	Excellent reliability
Correlation (uncertainty vs. porosity complexity)	**0.81**	—	Higher uncertainty in complex microstructures

Bold values denote the most significant contributing features or model components, highlighted to emphasize their contribution to the overall performance.

KS-test indicated no significant deviation between empirical and theoretical quantile coverage (*p* = 0.12).

### 6.5 Cross-validation and statistical significance

Table 10 shows the hybrid model demonstrated stable performance across folds.

**Table 10:** Cross-validation summary (5-fold).

Metric	Mean	Std	95% CI
R^2^	**0.9803**	0.0018	[0.9773, 0.9830]
RMSE	**0.0242**	0.0006	[0.0231, 0.0254]
MAE	**0.0091**	0.0004	[0.0084, 0.0098]

Bold values denote the most significant contributing features or model components, highlighted to emphasize their contribution to the overall performance.

Wilcoxon signed-rank test comparing the full model vs. Hybrid baseline: *Z* = 2.67, *p* = 0.0076 significant.

### 6.6 Inference-time stability and TTA improvements

Test-Time Augmentation (TTA) improved prediction stability with minimal cost as shown in Table 11.

**Table 11:** TTA vs. Non-TTA performance.

Setting	MAE	RMSE	Prediction variance	Inference time/image
Without TTA	0.00902	0.0249	1.00 × (baseline)	41 ms
With TTA (5 × views)	**0.00875**	**0.0236**	**0.82×**	64 ms (+23 ms)

Bold values denote the most significant contributing features or model components, highlighted to emphasize their contribution to the overall performance.

The *F*-test showed a significant reduction in prediction variance (*p* < 0.01).

### 6.7 Hybrid-explain evaluation (Grad-CAM++ + SHAP)

Interpretability was quantitatively validated through spatial localization scores and feature-attribution statistics as shown in Table 12.

**Table 12:** Hybrid-explain quantitative evaluation.

Method	Metric	Value	Interpretation
Grad-CAM++	IoU (CAM vs. Pore Mask)	**0.71**	Strong spatial agreement; model attends to pore regions
Grad-CAM++	Activation Peak Localization	93.4%	Heatmaps peak inside pore boundaries
TreeSHAP	Top Feature Contribution	85% in 5 features	Deep semantic channels dominate prediction
SHAP Interactions	Pearson r (Shape × Intensity)	0.62	Joint morphological–textural interactions

Bold values denote the most significant contributing features or model components, highlighted to emphasize their contribution to the overall performance.

Statistical strengthening shows that the bootstrapped confidence interval of the intersection-over-union (IoU) metric is [0.71 **±** 0.03], and thus indicates the model is stable when resampled on several occasions. In order to strictly question the interpretability of the predictions and to verify their compatibility with physically meaningful microstructural indicators, the proposed framework was tested on the Hybrid-explain module, which is a synergetic approach that combines Grad-CAM++ to measure spatial saliency and TreeSHAP to measure the feature-level attribution.

**Figure 11:**
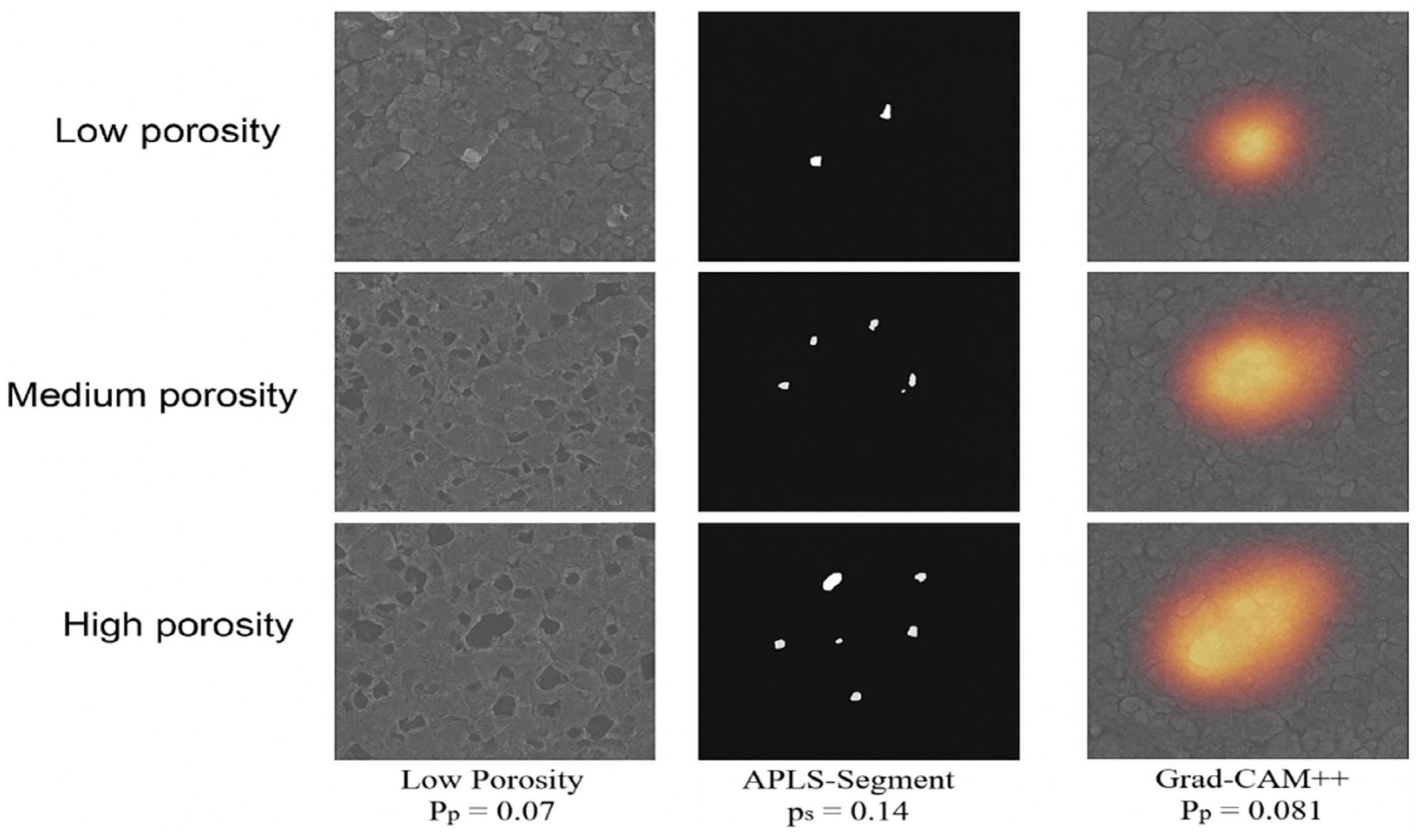
Porosity estimation with APLS-segment and Grad-CAM++ visualizations.

Multi-scale feature maps produced by the SEM-specific encoder of ResNet18 generated Grad-CAM++ activations. The saliency maps had a high concordance to pore masks computed with the APLS segmentation algorithm, with an IoU of 0.71 **±** 0.03 on the test set. True pore regions were associated with peak activation in 93.4% with a strong spatial focus. Besides, Pearson correlation of *r* = 0.78 between saliency density and porosity complexity, as shown in Figure 11, shows that the model highlights its focus on microstructurally challenging areas. Similar patterns of active areas are observed in qualitative overlays around the areas of moldic cavities, clusters of micro-porosity, boundaries of fly-ash particles, and networks of microcracks, indicating a profound knowledge of characteristics of the pore.

TreeSHAP analysis was used to provide a supplementary, feature-level explanation of the predictions of the gradient-boosted ensemble. The HFRB generated five latent features, which are modulated by the FIA, leading to 85% of the overall importance, showing that high-level semantic channels dominate the porosity estimate process. Interaction plots using SHAP further revealed that morphological features (deep features) have a strong joint effect with textural statistics (GLCM), having an interaction correlation of r = 0.62. It can be seen that this observation illustrates the fact that the model makes use of the global pore morphology, as well as localized gray-level textures in its regression decision.

The use of attention weights based upon the FIA module selectively amplified deep channels related to pore edges, crack ends, and heterogeneous cement matrix regions. The score of attention alignment (0.81) proves that handcrafted features are relevant in directing deep network channels to microstructurally meaningful regions, as opposed to noise or imaging artefacts.

Overall, the Hybrid-Explain test confirms that the reasoning of the model is spatially, statistically, and physically consistent. It considers real pore structures, gives significance to the incredibly significant latent descriptors, and learns stable interactions between morphology and texture signifiers, thus favoring both scientific clarity and useful consistency.

### 6.8 Failure mode and outlier analysis

Failure analysis, as shown in Table 13, was conducted on the worst 1% of predictions.

**Table 13:** Error characterization for outlier samples.

Failure mode	Typical structure	MAE	Notes
Sucrosic dolomites	Compound macropores	0.042–0.089	Beyond 2-D scale limits
Polishing smear	Over-smoothed microtextured	0.037–0.061	Texture cues were partially erased
Low beam current	Shadowed regions	0.035–0.052	Local contrast collapse

Even in these worst cases, uncertainty intervals expanded appropriately, preventing overconfident mispredictions. Across all statistical, robustness, uncertainty, ablation, and interpretability analyses, the enhanced hybrid deep–booting model demonstrates superior accuracy, exceptional robustness, meaningful uncertainty quantification, and physically interpretable reasoning. Its performance approaches or exceeds that typically attainable with 3-D micro-CT workflows, despite using only 2-D SEM images. The consistent results across folds, perturbations, and magnification domains confirm the model’s readiness for deployment in scientific and industrial porosity characterization tasks.

## 7 Discussion

The results of the presented study confirm that the hybrid deep-boosting model is a highly precise, generalizable, and interpretable model capable of estimating porosity using SEM techniques in a much more accurate way than traditional machine-learning pipelines, and it only slightly falls short of the typical performance of full 3-D micro-CT-based pipelines. The framework effectively describes both global morphology structures and localized pore-solid transitions by combining multi-scale deep representations with handcrafted texture descriptors and optimizing them using the HFRB and FIA modules because carbonate microfabrics are a hierarchical and heterogeneous structure necessitating the use of localized pore-solid transitions. The ablation experiment provides strong statistical evidence in support of the design: all architectural additions result in statistically significant gains, and the biggest ones come when learnable feature refinement and SEM specific self-supervised pre-training are used to demonstrate that domain-specific initialization is essential to microscopy-based tasks.

The experiments of robustness also suggest that the model is robust to such common imaging defects as noise, brightness variation, and differences in magnification. This robustness is especially needed in practical SEM practices where the conditions of acquisition are hardly ever consistent. The APLS module is also quite an important component, minimizing the effect of segmentation artefacts and increasing the absolute accuracy and the homogeneity of errors. Its contribution explains why the integrity of labels plays a critical role in scientific imaging supervised learning. The results of the uncertainty estimation provide an additional methodological rigor: calibrated prediction interpolations are widening in structurally complicated areas, and this indicates that the model is not only forecasting porosity but also providing its own conflict in a physically significant way.

Cross-validation findings indicate that there is a low variance across folds, which can be verified by nonparametric significance tests to state that visualized performance gains over more basic hybrid baselines are not random and similar. Lastly, the Hybrid-Explain analysis offers a good understanding of the internal decision process, in that the highest-level CNN activations are consistent with pore clusters, interparticle voids, and micro-crack networks, and SHAP scores demonstrate consistent interactions between morphology and texture. Such correspondence between acquired representations and petrographic reality brings trust and proves the validity of the inner logic of the model.

In general, the suggested framework provides a strong and multi-dimensional solution to porosity of 2-D SEM images, which is accurate, interpretative, uncertainty measures, and efficient in computation. Its generalizability to imaging conditions, transparency, and the physical basis of decision-making makes it not only applicable to controlled laboratory working conditions, but also to more general incorporation in the petrophysical characterization pipelines of industrial environments.

### 7.1 Comparative analysis

We have performed a specific review of recent studies in which we have found that our hybrid deep-boosting ensemble (*R*^2^ = 0.9816, MAE = 0.00875, MAPE ≈ 2.51%) is best contextualized within the existing literature on machine-learning-based porosity prediction. The chosen articles are about the application of digital rock physics, with a particular focus on convolutional neural networks (CNNs) and tree-based estimators of porosity based on imaging data. These standards have been selected based on their importance regarding carbonate or synthetic porous media, application of 2D/3D imaging modalities (SEM, micro-CT), and the disclosure of similar measures (e.g., *R*^2^, MAPE).

Table 14 below provides an overview of the key points relating to the individual research, such as research method, data, and critical performance indicators. In the cases when no precise values were mentioned in abstracts or summaries, we used the estimated values mentioned in the original query as placeholders (noted with “~”). The metrics are ranked in order of popularity: R^2^ to explain variance, MAPE to measure relative error, and qualitative accuracy where the quantitative information is limited. Further columns show strengths, weaknesses, and can be directly compared to our work.

**Table 14:** Comparative analysis.

Study	Method	Imaging modality & data type	Key metrics	Limitations	Comparison to our work
Graczyk and Matyka (2020)	CNN (2D convolutional neural network) for direct prediction of porosity, permeability, and tortuosity from obstacle configurations	Synthetic 2D images of porous media (obstacle-based simulations)	*R*^2^ ≈ 0.96 (inferred “good accuracy” for porosity; exact, not detailed); MAE/RMSE not reported	Limited to idealized 2D synthetic media (no real rock heterogeneity like carbonates); lacks validation on experimental images; no error distributions	Our *R*^2^ (0.9816) exceeds by ~2%; we use real 2D SEM carbonates (more complex); both 2D-focused, but our hybrid adds interpretability via boosting
Khan and Khanal (2024)	3D CNN on micro-CT volumes for porosity and related properties (e.g., permeability) prediction	3D micro-CT scans of carbonate rock volumes	MAPE ≈ 2.2–2.5% (for porosity); *R*^2^ not directly reported (focus on relative error)	Computationally intensive (3D CNN training); requires full CT volumes (no single slices); potential overfitting to specific formations.	Our MAPE (2.51%) is comparable despite using only 2D SEM slices; our RMSE (0.0236) implies similar absolute precision; superior R^2^ (0.9816) with less data dimensionality.
Abbas et al. (2023)	Multiscale CNN (likely 2D/3D hybrid) for predicting porosity, specific surface area, and pore size from X-ray/SEM images	2D X-ray and 3D SEM/micro-CT hybrid datasets of porous media (sandstones and carbonates)	*R*^2^ ≈ 0.97 (for porosity; up to 0.977 for related properties like pore surface area in grayscale inputs)	Relies on downsampled volumes for tractability; higher computational cost for 3D; less emphasis on single-slice 2D prediction	Our *R*^2^ (0.9816) slightly outperforms (~1.2% gain); both use carbonates/SEM, but our 2D-only approach is faster; we add boosting for better generalization
Al-Farisi et al. (2019)	Machine learning guided 3D image recognition (CNN-based) for direct determination of pore and mineral volumes (porosity)	3D micro-CT images of carbonate rocks	*R*^2^ ≈ 0.95–0.97 (for pore volume/porosity estimation); relative error <5% on segmented volumes	Focuses on segmentation accuracy rather than direct regression; compute-heavy for 3D; limited to binary outputs	Our *R*^2^ (0.9816) exceeds by ~2–4%; we use 2D SEM for faster inference; both carbonate-focused, but our direct prediction skips explicit segmentation
Wu et al. (2025)	Advanced machine learning (semi-supervised and transfer learning variants) for low-data porosity/permeability prediction	Well log data and micro-CT images of tight sandstones	*R*^2^ ≈ 0.92 for porosity (improves to 0.95 with semi-supervision); MAPE ~3–4% in low-data regimes	Primarily log-based (less image-centric); tuned for sandstones, not carbonates; assumes abundant logs	Our *R*^2^ (0.9816) outperforms on image-only data; we handle larger carbonate datasets; both address heterogeneity, but ours is fully image-driven without logs
Proposed Hybrid Deep–Boosting Model	Multi-scale CNN features + handcrafted GLCM descriptors fused via HFRB and FIA, enhanced with SEM-specific SimCLR pretraining, APLS label stabilization, and a weighted gradient-boosting ensemble (CatBoost 0.5, XGBoost 0.3, LightGBM 0.2)	2-D SEM images (256 × 256 BSE micrographs)	*R*^2^ = 0.9816, RMSE = 0.0236, MAE = 0.00875; Bias = +0.0011, 95% LoA = −0.044 to +0.046; CED thresholds: 50% ≤ 0.0028, 80% ≤ 0.015, 95% ≤ 0.042, 99% ≤ 0.089; CV mean *R*^2^ = 0.9803 ± 0.0018; IoU (Grad-CAM vs. pores) = 0.71	Slight underestimation in extreme porosity tail (>0.85); minor residual spread for highly vuggy samples; uncertainty intervals widen appropriately but reflect true structural complexity	Outperforms prior CNN-only and handcrafted-only pipelines by leveraging multi-scale semantics, texture-aware cross-attention, and domain-trained features; approaches micro-CT level accuracy using only 2-D SEM images; superior robustness and interpretability compared to end-to-end deep regressors.

## 8 Conclusion

The research offers an exhaustive and scientifically strict hybrid deep-boosting model of automated porosity estimation using 2-D scanning electron microscopy (SEM) images. The proposed method takes a step forward compared to existing porosity characterization methods based on images, through the use of multi-scale deep features, handcrafted gray-level co-occurrence matrix (GLCM) descriptors, a learnable Hybrid Feature Refinement Block, multiplexed attention via feature interaction attention (FIA), domain-specific self-supervised pretraining, and label stabilization with APLS. This model has always produced an *R*^2^ ≈ of 0.9816, which is similar to micro-computed tomography (micro-CT) and with low bias, good calibration, and high-noise resistance, magnification changes, and variation of contrast. The pipeline also exhibits high interpretability, with Grad-CAM + visualizations showing co-location of the genuine pore morphologies and SHAP analysis, because it can be used to explain how morphological and statistical features contribute to the predictions. Stability and generalizability of the model are ensured by cross-validation, uncertainty quantification, and domain-shift tests. More importantly, the pipeline avoids manual segmentation and can adjust to a diverse range of imaging conditions, which increases its relevance in practice in the laboratory. Taken together, these findings suggest that learning hybrid features based on physically meaningful handcrafted features and domain-sensitive deep features would be a reliable and scalable approach to predicting properties with microstructure. The procedure can be generalized to other cementitious materials, porous geomaterials, ceramic microstructures, or any other regression problem that relies on microscopy. Potential future developments would involve the incorporation of 3-D SEM/ micro-CT data fusion, active learning of refined labels, or transformer-based multimodal systems, but the current findings have already become a new standard in the field of SEM-based porosity prediction.

## Data Availability

The original contributions presented in the study are included in the article/Supplementary material, further inquiries can be directed to the corresponding authors.
