# Supplementary material for: A hybrid deep boosting framework with adaptive label stabilization for SEM-based porosity estimation in fly-ash cement mortar
Source: Front Artif Intell. 2026 Feb 23;9:1766671. doi: 10.3389/frai.2026.1766671 (PMC12968296; doi:10.3389/frai.2026.1766671)
Supplement: Supplementary file 1 [file Data_Sheet_1.docx]

Appendix A

Table A1: Deep Feature Settings

| **Component** | **Setting** |
| --- | --- |
| Backbone | ResNet-18 (SEM SimCLR-pretrained) |
| Fine-tuned layers | Layer 3 and Layer 4 |
| Multi-scale features | 256-D (Layer 3) + 512-D (Layer 4) = 768-D |
| Input resolution | 256×256 grayscale |
| Normalization | SEM dataset mean/variance |

Table A2: Handcrafted Feature Settings

| **Component** | **Setting** |
| --- | --- |
| GLCM distances | 1 |
| GLCM angles | 0°, 45°, 90°, 135° |
| Gray levels | 256 |
| Features (7) | Pore fraction (APLS), Contrast, Dissimilarity, Homogeneity, Energy, Correlation, Intensity |
| Combined feature space before fusion | 768-D + 7-D = 775-D |

Table A3: HFRB and FIA Hyperparameters

| **Component** | **Setting** |
| --- | --- |
| HFRB input | 775-D |
| HFRB hidden/output | 256 → 128-D |
| Activation | GELU |
| LayerNorm ε | 1e-5 |
| Dropout | 0.2 |
| FIA heads | 4 |
| K/Q/V dimension | 32 |
| FIA dropout | 0.1 |

Table A4: APLS + Uncertainty Parameters

| **Component** | **Setting** |
| --- | --- |
| Threshold search | ±10% of Totsu​ |
| Entropy threshold | 0.65– 0.75 |
| Morphological weight λ | 0.1 |
| Soft-label weighting | Gaussian decay |
| MC-Dropout passes | 50 |
| Quantile outputs | 0.05, 0.50, 0.95 |

Table A5: Training and Inference Settings

| **Component** | **Setting** |
| --- | --- |
| SSL epochs | 200 |
| SSL optimizer | AdamW (lr = 1e-3, cosine) |
| Device | CUDA GPU |
| Seeds | Python/NumPy/PyTorch = 42 |
| TTA augmentations | 5 (flips, rotations, ±5% scaling, contrast jitter) |
| Final prediction | Mean of TTA outputs |

Table A6: Persistence Settings

| **Component** | **Setting** |
| --- | --- |
| Storage format | joblib |
| Filename | porosity_hybrid_deep_boosting.pkl |
| Stored components | SSL encoder, HFRB, FIA, boosting models, ensemble |
